# Analytic solutions for Euler–Bernoulli beams with axial compression resting on a nonlinear elastic foundation using MADM

**DOI:** 10.1038/s41598-026-41700-2

**Published:** 2026-03-03

**Authors:** Li-Kuo Chou, Ming-Xian Lin

**Affiliations:** 1 College of Optoelectronic Manufacturing, Zhejiang Industry & Trade Vocational College, Wenzhou, 325003 Zhejiang Province China; 2 Department of Mechanical and Computer-Aided Engineering, Feng Chia University, Taichung, Taiwan

**Keywords:** Euler–Bernoulli beams, Modified Adomain decomposition method, Nonlinear elastic foundation beam, Axial compression, Engineering, Mathematics and computing, Physics

## Abstract

This article investigates the deflection behavior of Euler–Bernoulli beams subjected to axial compression and resting on a nonlinear elastic foundation. The Modified Adomian Decomposition Method (MADM) is employed to predict the beam deflection under various loading and foundation conditions. By adopting an initial polynomial ansatz, MADM effectively optimizes the construction of Adomian polynomials, resulting in rapid convergence and an accurate series solution. The accuracy and reliability of the proposed method are examined through two illustrative cases. In the first case, the formulation is verified against an analytically tractable benchmark problem, while in the second case, the MADM results are compared with previously published solutions. The comparative analysis demonstrates excellent agreement with existing studies, confirming the validity of the proposed approach. Overall, the results indicate that MADM provides a stable and efficient analytical framework for modeling the coupled effects of axial compression and nonlinear elastic foundations in Euler–Bernoulli beams.

**Supplementary Information:**

The online version contains supplementary material available at 10.1038/s41598-026-41700-2.

## Introduction

The theory of elastic structures, particularly beams and plates resting on elastic foundations, plays a central role in modern structural mechanics due to its wide applications in helicopter blades, turbine blades, railway systems, and bridge structures. Since the early development of elastic foundation theory, extensive studies have focused on formulating governing differential equations and boundary conditions to investigate static deflection and vibration responses under various loading scenarios^1–3^. Systematic validation has been shown to improve modeling accuracy and stability^4–6^. When analyzing beams on elastic foundations subjected to uniformly distributed loads, researchers generally derive the governing differential equations along with appropriate boundary conditions to determine the beam deflection and vibration characteristics. Numerical values are then obtained through various mathematical formulations, focusing on both static and dynamic responses. Understanding the relationship between the governing variables in these equations is crucial for accurately predicting vibration frequencies and static deflections, which are fundamental for reliable engineering applications.

Beams are often modeled with uniform geometries^7–18^, and structural analysis is typically performed in a systematic and sequential manner. The Euler–Bernoulli and Timoshenko beam theories remain the classical foundations for analyzing beam behavior, and many studies have rigorously examined their accuracy and applicability^5,9,10,13,15,18,19^. A wide variety of analytical and numerical techniques have been developed to address linear and nonlinear beam problems on elastic foundations. These include the perturbation method^4^, shift function approach^8^, finite element method (FEM)^19^, Adomian decomposition method (ADM)^20–22^, and differential transform method (DTM)^23–28^. These methods provide flexible mathematical tools for solving complex beam–foundation interactions, covering a wide range of loading, boundary, and material conditions.

In the past five years, many scholars have applied various analytical and numerical methods to investigate the behavior of beams on elastic foundations. Zhang et al.^29^ compared Euler–Bernoulli and Timoshenko beam formulations to study the effects of moving loads on periodically supported beams. Their results showed that for a moving constant load expressed as an equivalent surface roughness, the influence of load speed is negligible until the sleeper passing frequency approaches the vertical resonance of the track, where the track mass begins to bounce on the support stiffness. Xu et al.^30^ analyzed the transverse free vibration of an Euler–Bernoulli beam with pre-axial pressure resting on a variable Pasternak elastic foundation under arbitrary boundary conditions. They solved the governing differential equation containing nonlinear partial derivative terms of the shape function by constructing a system of stiffness equations composed of obtained matrices, instead of relying on a single equation based on classical beam theory. Doeva et al.^31^ performed a static analysis of composite beams on variable stiffness elastic foundations using the Homotopy Analysis Method (HAM). Their results were compared with both published literature and the Chebyshev Collocation Method to confirm the validity and accuracy of the proposed approach. Luo et al.^32^ developed an exact closed-form solution for the free vibration of Euler–Bernoulli and Timoshenko beams with intermediate elastic supports. The accuracy of their solution was verified using numerical simulations based on the Finite Element Method (FEM) and validated through comparisons with results obtained by the Transfer Matrix Method (TMM) and Green’s Function Method (GFM).

Hadji et al.^33^ investigated the bending and free vibration of porous functionally graded (PFG) beams resting on elastic foundations. They adopted hyperbolic shear deformation theory to formulate kinematic relations and derived the equations of motion using Hamilton’s principle. Kanwal et al.^34^ examined the influence of shear deformation and rotary inertia on elastically constrained beams supported by Pasternak foundations. Their findings demonstrated the precision and efficiency of the Finite Element Method through comparisons with analytical results for both general and special cases. Olotu et al.^35^ performed a free vibration analysis of tapered Rayleigh beams on variable two-parameter elastic foundations and explored the effects of Pasternak foundation variation on natural frequencies for different slenderness ratios. Mellal et al.^36^ analyzed the vibration and buckling behavior of porous functionally graded (FG) beams resting on variable elastic foundations using higher-order shear deformation theory. They derived the equations of motion through Hamilton’s principle and obtained analytical solutions using Navier’s method for simply supported FG beams. Wu et al.^37^ proposed a simple and unified mesh-free approach to develop arbitrary-order Hermite shape functions for Euler–Bernoulli beam elements. This formulation provides an explicit framework for constructing higher-order Hermite beam elements efficiently. Zemskov et al.^38^ addressed the problem of unsteady vibrations in Euler–Bernoulli beams by considering relaxation effects in thermal and diffusion processes. The governing equations were derived from the general model of thermoelastic diffusion in a continuum using the variational D’Alembert principle, providing valuable insight into the coupled thermo-mechanical behavior of beam structures.

Recent studies have addressed nonlinear and coupled behaviors in structural and mechanical systems using analytical, numerical, experimental, and data-driven approaches. Zhu et al.^39^ proposed a quantum interval neural network framework to address high-dimensional uncertainty in structural statics, highlighting the need for efficient computational strategies. Singh et al.^40^ investigated thermoelastic damping and frequency shifts in Euler–Bernoulli beams under various boundary conditions, while Qi et al.^41^ developed an analytical–numerical model to capture axial–flexure–shear interaction in reinforced concrete walls, emphasizing the importance of accurately representing coupled nonlinear mechanisms in load-bearing structures. Beyond beam-type structures, nonlinear mechanical behavior has also been studied in related engineering systems. Li and Ma^42^ examined the combined influence of stress path and inherent anisotropy on mechanical behavior under complex stress states, demonstrating strong nonlinearity and path dependence in geomaterials. Liu et al.^43^ presented an analytical and experimentally validated framework for characterizing coupled vibration behavior in hemispherical resonators with mass defects, revealing the impact of higher-order nonlinearities on dynamic response and system stability. In infrastructure-related applications, Chen et al.^44^ investigated thermochromic composite coatings for asphalt pavements under extreme thermal loading, highlighting the need to control deformation and stress responses induced by non-uniform thermal effects.

In parallel, high-fidelity numerical simulations and data-driven techniques have been widely adopted to analyze complex deformation and interaction problems. Huang et al.^45^ conducted finite element analyses of reinforced concrete columns under combined axial compression, torsion, and bending, showing that axial load ratios and eccentricity significantly influence stiffness, ductility, and energy dissipation. Yin et al.^46^ developed an analytical framework to evaluate tunnel deformation induced by foundation pit excavation, demonstrating the dependence of structural response on soil stiffness and geometric parameters. Zhang and Zhang^47^ examined viscoelastic impact mitigation between adjacent buildings considering soil–structure interaction, showing that nonlinear material behavior and interaction effects play a critical role in dynamic response control. Recent machine learning–assisted approaches, such as those proposed by Hu et al.^48^ and Li et al.^49^, demonstrated the capability of data-driven models to predict ground and pipeline settlement under complex construction environments; however, these approaches typically require extensive computational resources or training data and provide limited analytical insight into the governing mechanics. Recent work has also emphasized nonlinear restoring mechanisms and stabilization strategies in flexible structures. Zou et al.^50^ investigated customized nonlinear force mechanisms for vibration mitigation, while Hu et al.^51^, Dai et al.^52^, and Gai et al.^53^ demonstrated the effectiveness of advanced damping and control strategies for suppressing vortex-induced vibrations in long-span bridges. In addition, Yang et al.^54^ proposed a measurement-based adaptive framework to improve the reliability of finite element modeling for structures with uncertain subcomponents, and Li et al.^55^ highlighted the nonlinear and stress-history-dependent behavior of organic soils, indicating that foundation stiffness and deformation characteristics can evolve nonlinearly under loading. These studies collectively suggest that modeling uncertainty and nonlinear foundation behavior can substantially influence structural response and stability. Building on closely related studies on Euler–Bernoulli beams and elastic foundations^16,22,24^, the present work develops a semi-analytical MADM-based framework for Euler–Bernoulli beams resting on nonlinear elastic foundations under axial compression. Unlike purely numerical or data-driven approaches, the proposed method directly solves the nonlinear governing equations without assuming weak nonlinearity, enabling efficient investigation of axial-force effects and nonlinear foundation behavior with clear physical interpretability.

In summary, recent studies have considerably advanced the theoretical and computational understanding of beams resting on elastic foundations, yet most existing analytical and numerical approaches, including the finite element method, perturbation techniques, and transform-based solutions, still involve complicated formulations and demand significant computational effort, particularly when analyzing nonlinear foundation behavior under compressive or dynamic loads. Moreover, only limited attention has been given to the convergence stability and efficiency of analytical series solutions for nonlinear elastic foundation system. To address these limitations, this study employs the MADM to analyze the compressive deflection and convergence characteristics of beams on nonlinear elastic foundations. The proposed method establishes a simplified analytical framework that maintains high accuracy while effectively capturing nonlinear mechanical responses. Two illustrative examples are presented to validate the reliability of the approach. All symbolic derivations and numerical computations are implemented using Maple software, confirming that the Modified Adomian Decomposition Method provides accurate, stable, and computationally efficient solutions for nonlinear beam–foundation problems in engineering applications.

## Mathematical model of the modified Adomian decomposition method

At the beginning of this section, the distinction between the classical Adomian Decomposition Method (ADM) and the Modified Adomian Decomposition Method (MADM) is clarified. In classical ADM, the solution is expressed as a series and the nonlinear terms are decomposed using Adomian polynomials derived directly from the original nonlinear operator. In contrast, MADM selects the highest-order differential operator as the linear operator and adopts an initial polynomial ansatz to improve the construction of Adomian polynomials, leading to enhanced convergence and numerical stability for strongly nonlinear problems. Originally proposed by Adomian^56^, MADM provides a straightforward, efficient, and reliable analytical framework for solving nonlinear differential equations. Within this framework, the governing differential equation is formulated in the following general form:

1 $$Fu\left( x \right) = Lu\left( x \right) + Ru\left( x \right) + Nu\left( x \right) = g\left( x \right).$$

In this formulation, $F(u)$ consists of linear and nonlinear components, where $L\left(u\right)+R(u)$ represents the linear part and $N(u)$ denotes the nonlinear differential operator. Here, $u$ is the unknown physical quantity, $L$ is the highest-order linear differential operator, and $R$ contains the remaining linear terms.

Although the choice of the invertible operator $L$ is theoretically arbitrary, selecting the highest-order differential operator facilitates the evaluation of ${L}^{-1}$ by avoiding complications associated with Green’s functions. Accordingly, Eq. (1) can be rewritten as follows:

2 $$Lu\left( x \right) = g\left( x \right) - Ru\left( x \right) - Nu\left( x \right).$$

Given that *Lu*(*x*) is an invertible linear operator, Eq. (2) can be re-expressed as

3 $$L^{{ - {1}}} Lu\left( x \right) = L^{{ - {1}}} g\left( x \right) - L^{{ - {1}}} Ru\left( x \right) - L^{{ - {1}}} Nu\left( x \right).$$

The operator *L*^−1^*u*(*x*) is defined as the definite integral from 0 to *x*. Suppose that *L* is an *n-*th order differential operator, where *L* = $\frac{{d}^{n}}{d{x}^{n}}$, then *L*^−1^ is an *n*-th order integral operator. For boundary value problems, *L*^−1^ represents an indefinite integral, and the integration constant Φ(*x*) is determined by the boundary conditions. Therefore, Eq. (3) can be written as

4 $$u\left( x \right) = L^{{ - {1}}} g\left( x \right) - L^{{ - {1}}} Ru\left( x \right) - L^{{ - {1}}} Nu\left( x \right) + \Phi \left( x \right)$$

Herein, the function Φ(*x*) satisfies *L*Φ(*x*) = 0. In this example, we suppose that *L* is a second-order differential operator. Thus, Φ(*x*) can be defined as follows

5 $$\Phi \left( x \right) = u\left( 0 \right) + x\left( {\left. {\frac{{du(x)}}{{dx}}} \right|_{{x = 0}} } \right)$$

By substituting Eq. (5) into Eq. (4), *u*(*x*) can be expressed as

6 $$u\left( x \right) = L^{{ - 1}} g(x) - L^{{ - 1}} Ru(x) - L^{{ - 1}} Nu(x) + u(0) + x\left( {\left. {\frac{{du(x)}}{{dx}}} \right|_{{x = 0}} } \right)$$

In addition to decomposing the operator and finding its inverse operator, the Adomian Decomposition Method (ADM) expresses *u*(*x*) as an infinite sum of functions.

7 $$u\left(x\right)=\sum_{n=0}^{\infty }{u}_{n}(x)={u}_{1}\left(x\right)+{u}_{2}\left(x\right)+{u}_{3}\left(x\right)+{u}_{4}\left(x\right)+\cdots$$

Furthermore, if the nonlinear term *Nu*(*x*) can be written as *f*(*u*(*x*)), a function of *u*(*x*), then *Nu*(*x*) can be decomposed as follows

8 $$Nu\left(x\right)=f(u(x))=\sum_{n=0}^{\infty }{A}_{n}(x)={A}_{1}\left(x\right)+{A}_{2}\left(x\right)+{A}_{3}\left(x\right)+{A}_{4}\left(x\right)+\cdots$$

In this decomposition, *A*_*n*_ denote the Adomian polynomials.

9 $${A}_{0}=f({u}_{0})$$

10 $${A}_{1}={u}_{1}{f}^{(1)}({u}_{0})$$

11 $${A}_{2}={u}_{2}{f}^{\left(1\right)}\left({u}_{0}\right)+\frac{1}{2!}{u}_{1}^{2}{f}^{\left(2\right)}({u}_{0})$$

12 $${A}_{3}={u}_{3}{f}^{\left(1\right)}\left({u}_{0}\right)+{u}_{1}{u}_{2}{f}^{\left(2\right)}({u}_{0})+\frac{1}{3!}{u}_{1}^{3}{f}^{\left(3\right)}({u}_{0})$$

$$\vdots$$

13 $${A}_{n}=\frac{1}{n!}\left[\frac{{d}^{n}}{{dx}^{n}}f({u}_{0}(x))\right], n=\mathrm{0,1},2,\cdots$$

By substituting Eqs. (7) and (8) into Eq. (6), the following result is obtained.

14 $$\sum_{n=0}^{\infty }{u}_{n}(x)=u\left(0\right)+x\left({\left.\frac{du(x)}{dx}\right|}_{x=0}\right)+{L}^{-1}g\left(x\right)+{L}^{-1}r\sum_{n=0}^{\infty }{u}_{n}\left(x\right)-{L}^{-1}\sum_{n=0}^{\infty }{A}_{n}(x)$$

After expanding Eq. (14), the following recursive relation is obtained

15 $${u}_{0}(x)=u\left(0\right)+x\left({\left.\frac{du(x)}{dx}\right|}_{x=0}\right)+{L}^{-1}g\left(x\right)$$

16 $${u}_{n}\left(x\right)=-{L}^{-1}R\sum_{n=0}^{\infty }{u}_{n-1}\left(x\right)-{L}^{-1}\sum_{n=0}^{\infty }{A}_{n-1}\left(x\right), n\ge 1$$

From Eq. (16), it can be seen that each *u*_*n*_(*x*) depends only on *u*_*n*−1_(*x*) and its corresponding Adomian polynomials, and *u*_0_(*x*) is expressed solely in terms of the initial conditions. Hence, once the Adomian polynomials are determined, each *u*_*n*_(*x*) can be calculated. Finally, an infinite series solution can be obtained.

17 $${u}_{n}\left(x\right)=\sum_{n=0}^{\infty }{u}_{n}(x)$$

A key feature of MADM lies in the deliberate choice of the linear operator $L$ as the highest-order differential operator in the governing equation. This choice allows the inverse operator ${L}^{-1}$ to be evaluated analytically and avoids the use of Green’s functions, thereby reducing truncation-induced oscillations and improving convergence stability compared with the classical ADM formulation. Moreover, MADM directly handles both linear and nonlinear terms without resorting to linearization, which contributes to the rapid convergence of the resulting series solution.

In Eq. (17), as *n* → ∞, the numerical solution becomes increasingly accurate. In practice, a partial sum of the series of polynomials is chosen to meet accuracy requirements. Numerical solutions obtained via the MADM converge rapidly and attain high accuracy. To provide a concrete convergence metric, we evaluate the successive-difference error of the deflection V(N) as *E*(*N*) = ∣*V*(*N*) − *V*(*N* − 1)∣. denotes the deflection obtained from the N-term MADM approximation. With the prescribed tolerance E(N) ≤ 1 × 10^−10^, the MADM series exhibits rapid decay of E(N) and readily satisfies this criterion, demonstrating its high accuracy and fast convergence for the nonlinear problem.

## Mathematical modeling of the Euler–Bernoulli beams

**Fig. 1:**
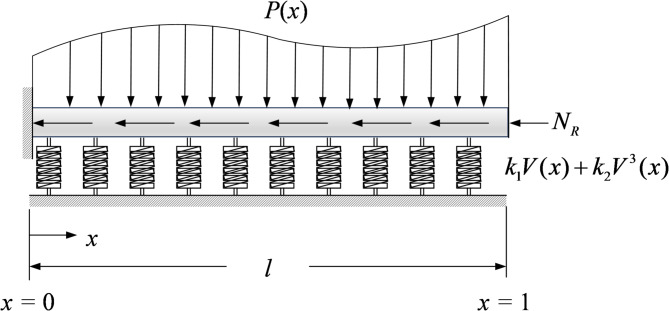
A schematic diagram of an Euler–Bernoulli beam resting on a nonlinear elastic foundation subjected to axial compressive and transverse loading.

Consider the deflection of Euler–Bernoulli beams on linear and nonlinear elastic foundation subjection to axial compression and transverse distributed force shown in Fig. 1 is taken into consideration in this study.

According to the formulation presented by Olotu et al.^35^, the static flexural displacement $\overline{V }\left(X\right)$ of the beam is governed by the following ordinary differential equation:

18 $$\frac{{d}^{2}}{d{X}^{2}}\left(E(X)I(X)\frac{{d}^{2}\overline{V }(X)}{{dX}^{2}}\right)-\frac{d}{dX}\left({N}_{R}(X)\frac{d\overline{V }(X)}{dX}\right)+{K}_{1}\left(X\right)\overline{V }\left(X\right)+{K}_{2}\left(X\right){\overline{V} }^{3}\left(X\right)=p(X)$$

Here, $X$ denotes the coordinate along the beam, and $l$ represents the total beam length. The material and foundation properties may vary with position and are described by the Young’s modulus $E(X)$, moment of inertia $I(X)$, linear foundation stiffness ${K}_{1}(X)$, and nonlinear foundation stiffness ${K}_{2}(X)$. The linear foundation stiffness ${K}_{1}(X)$ characterizes the initial elastic response of the supporting medium, consistent with a Winkler-type foundation model, whereas the nonlinear stiffness ${K}_{2}(X)$ represents displacement-dependent restoring behavior arising from material nonlinearity, contact effects, or geometric constraints in practical foundation systems. In soil-supported structures, this nonlinear response is commonly associated with stress-dependent stiffness, particle rearrangement, and progressive mobilization of soil resistance, which cause the foundation reaction to deviate from a linear force–displacement relationship as deformation increases. The axial compressive force is expressed as ${N}_{R}(X)$, and the transverse load is given by $p(X)$. The boundary conditions are specified as follows:

19 $$\overline{V }\left(0\right)=0,\overline{{V }{\prime}}\left(0\right),\overline{{V }^{{\prime}{\prime}}}\left(1\right),\overline{{V }^{{\prime}{\prime}{\prime}}}\left(l\right)-{N}_{R}\overline{{V }{\prime}}\left(l\right)=0$$

The nondimensional parameters for the Euler–Bernoulli beam on an elastic foundation subjected to axial compression and transverse force are defined as follows

20 $$x=\frac{X}{l},V=\frac{\overline{V }\left(X\right)}{l},P\left(x\right)=\frac{p\left(X\right){l}^{3}}{E\left(X\right)I\left(X\right)},{k}_{1}\left(x\right)=\frac{{K}_{1}\left(X\right){l}^{4}}{E\left(X\right)I\left(X\right)},{k}_{2}\left(x\right)=\frac{{K}_{2}\left(X\right){l}^{6}}{E\left(X\right)I\left(X\right)},r(x)=\frac{{-N}_{R}(X){l}^{2}}{E(X)I(X)}$$

By substituting Eq. (20) into Eq. (19), the governing differential equation can be written in the following nondimensional form:

21 $$\frac{{d}^{4}V}{d{x}^{4}}+r\frac{{d}^{2}V}{d{x}^{2}}+{k}_{1}V+{k}_{2}{V}^{3}=P$$

and the associated boundary conditions become

22 $$V\left(0\right)=0,{V}^{{\prime}\left(0\right)},{V}^{{\prime}{\prime}\left(1\right)},V{\prime}{\prime}{\prime}\left(1\right)-rV{\prime}\left(1\right)=0$$

The boundary conditions correspond to a clamped–free (cantilever) Euler–Bernoulli beam. At $X=0$, $V(0)=0$ and $V^{\prime } \left( 0 \right) = 0$ enforce zero transverse displacement and zero rotation (clamped end). At $X = 1$, $V^{\prime \prime } \left( 1 \right) = 0$ imposes a zero bending moment (free end), and $V^{\prime \prime \prime } \left( 1 \right) - rV^{\prime } \left( 1 \right) = 0$ represents the zero effective shear-force condition in the presence of an axial force.

Here, $V$ denotes the transverse deflection, and $x$ represents the spatial coordinate along the beam. The parameter ${k}_{1}$ is the linear elastic foundation modulus, ${k}_{2}$ is the nonlinear elastic foundation modulus, $r$ refers to the axial compressive force, and $P$ denotes the applied transverse load per unit length. When the applied distributed load and axial compressive force are analytic functions, the deflection $V\left(x\right)$ and the load $P\left(x\right)$ can be expressed using the following Maclaurin series expansions.

23 $$V\left(x\right)=\sum_{m=0}^{\infty }{a}_{m}{x}^{m}$$

24 $$P\left(x\right)=\sum_{m=0}^{\infty }{P}_{m}{x}^{m}$$

Hence

25 $$\frac{dV}{dx}=\sum_{m=0}^{\infty }(m+1){a}_{m+1}{x}^{m}$$

26 $$\frac{{d}^{2}V}{d{x}^{2}}=\sum_{m=0}^{\infty }(m+1)(m+2){a}_{m+2}{x}^{m}$$

27 $$\frac{{d}^{3}V}{d{x}^{3}}=\sum_{m=0}^{\infty }(m+1)(m+2)(m+3){a}_{m+3}{x}^{m}$$

28 $$\frac{{d}^{4}V}{d{x}^{4}}=\sum_{m=0}^{\infty }(m+1)(m+2)(m+3)(m+4){a}_{m+4}{x}^{m}$$

The nonlinear term ${V}^{3}$(*x*) can be expressed in the following form

29 $${V}^{3}\left(x\right)=\left\{\sum_{n=0}^{\infty }{a}_{n}{x}^{n}\right\}\left\{\sum_{v=0}^{\infty }{a}_{v}{x}^{v}\right\}\left\{\sum_{\mu =0}^{\infty }{a}_{\mu }{x}^{\mu }\right\}=\sum_{\mu =0}^{\infty }{A}_{m}({a}_{0},\cdots ,{a}_{m}){x}^{m}$$

where the coefficients of Adomian polynomials are

30 $${A}_{m}\left({a}_{0},\cdots ,{a}_{m}\right)=\sum_{v=0}^{m}\sum_{\mu =0}^{v}{a}_{m-v}{a}_{v-\mu }$$

For convenience, some coefficients of the Adomian polynomials corresponding to the nonlinear terms are listed below.

31 $${A}_{0}={a}_{0}^{3}$$

32 $${A}_{1}=3{a}_{0}^{2}{a}_{1}$$

33 $${A}_{2}=3{a}_{0}{a}_{1}^{2}+3{a}_{0}^{2}{a}_{2}$$

34 $${A}_{3}=3{a}_{0}^{2}{a}_{3}+{a}_{1}^{3}+6{a}_{0}{a}_{1}{a}_{2}$$

35 $${A}_{4}=3{a}_{0}^{2}{a}_{4}+3{a}_{1}^{2}{a}_{2}+6{a}_{0}{a}_{1}{a}_{3}+3{a}_{0}{a}_{2}^{2}$$

36 $${A}_{5}=3{a}_{1}^{2}{a}_{3}+3{a}_{1}{a}_{2}^{2}+3{a}_{0}^{2}{a}_{5}+6{a}_{0}{a}_{1}{a}_{4}+6{a}_{0}{a}_{2}{a}_{3}$$

37 $${A}_{6}=3{a}_{0}{a}_{3}^{2}+6{a}_{0}{a}_{1}{a}_{5}+6{a}_{1}{a}_{2}{a}_{3}+3{a}_{1}^{2}{a}_{4}+6{a}_{0}{a}_{2}{a}_{4}+{a}_{2}^{3}+3{a}_{0}^{2}{a}_{6}$$

38 $${A}_{7}=3{a}_{1}{a}_{3}^{2}++6{a}_{1}{a}_{2}{a}_{4}+6{a}_{0}{a}_{3}{a}_{4}+3{a}_{2}^{2}{a}_{3}+3{a}_{0}^{2}{a}_{7}+6{a}_{0}{a}_{2}{a}_{5}+3{a}_{1}^{2}{a}_{5}$$

39 $${A}_{8}=6{a}_{1}{a}_{2}{a}_{5}+6{a}_{0}{a}_{3}{a}_{5}+3{a}_{2}^{2}{a}_{4}+3{a}_{0}{a}_{4}^{2}+6{a}_{0}{a}_{2}{a}_{6}+3{a}_{2}{a}_{3}^{2}+3{a}_{0}^{2}{a}_{8}+6{a}_{1}{a}_{3}{a}_{4}6{a}_{0}{a}_{1}{a}_{7}+3{a}_{1}^{2}{a}_{6}$$

40 $$\begin{aligned} A_{9} = & 3a_{1} a_{4}^{2} + 3a_{0}^{2} a_{9} + 6a_{1} a_{2} a_{6} + 6a_{0} a_{1} a_{8} + 6a_{0} a_{4} a_{5} + 3a_{2}^{2} a_{5} + a_{3}^{3} \\ & + 6a_{2} a_{3} a_{4} 6a_{1} a_{3} a_{5} + 6a_{0} a_{2} a_{7} + 3a_{2}^{1} a_{7} + 6a_{0} a_{3} a_{6} \\ \end{aligned}$$

41 $$\begin{aligned} A_{{10}} = & 3a_{0} a_{5}^{2} + 3a_{3}^{2} a_{4} + 6a_{1} a_{2} a_{7} + 6a_{0} a_{3} a_{7} + 6a_{2} a_{3} a_{5} + 3a_{0}^{2} a_{{10}} + 6a_{0} a_{4} a_{6} 3a_{2}^{2} a_{6} \\ & + 3a_{1}^{2} a_{8} + 6a_{0} a_{1} a_{9} + 3a_{2} a_{4}^{2} + 6a_{0} a_{2} a_{8} + 6a_{1} a_{4} a_{5} + 6a_{1} a_{3} a_{6} \\ \end{aligned}$$

$$\begin{gathered} \vdots \hfill \\ A_{n} \hfill \\ \end{gathered}$$

By substituting Eqs. (23) through (29) back into Eq. (21)

42 $$\begin{aligned} & \sum\limits_{{m = 0}}^{\infty } {\left( {m + 1} \right)} \left( {m + 2} \right)\left( {m + 3} \right)\left( {m + 4} \right)a_{{m + 4}} x^{m} \\ & \quad \quad + r\sum\limits_{{m = 0}}^{\infty } {\left( {m + 1} \right)} \left( {m + 2} \right)a_{{m + 2}} x^{m} + k_{1} \sum\limits_{{m = 0}}^{\infty } {a_{m} } x^{m} \\ & \quad \quad + k_{2} \sum\limits_{{m = 0}}^{\infty } {A_{m} } \left( {a_{0} , \cdots ,a_{n} } \right)x^{m} = \sum\limits_{{m = 0}}^{\infty } {P_{m} } x^{m} \\ \end{aligned}$$

Once the coefficients of the power series have been collected, the following recurrence formula is obtainable.

43 $${a}_{m+4}=\frac{{P}_{m}+R\left(m+1\right)\left(m+2\right)-{k}_{1}{a}_{m}-{k}_{2}{A}_{m}}{(m+1)(m+2)(m+3)(m+4)}$$

From the recurrence relation together with Eq. (30), it follows that all coefficients ${a}_{m}$ can be expressed in terms of four unknowns, namely ${a}_{0}$, ${a}_{1}$, ${a}_{2}$, and ${a}_{3}$. These four coefficients are determined by enforcing the boundary conditions specified in Eq. (22). Consequently, every coefficient ${a}_{m}$ in the Maclaurin expansion of the deflection function $V(x)$ can be evaluated.

In the numerical implementation, only a finite number of terms in the series is retained. When $n+1$ terms are used, the approximate deflection is given by

44 $$V(x)=\sum_{m=0}^{n}{a}_{m}{x}^{m}$$

and the coefficients of Adomain polynomial are

45 $${A}_{n}\left({a}_{0},\cdots ,{a}_{n}\right)=\sum_{v=0}^{n}\sum_{\mu }^{v}{a}_{n=v}{a}_{v-\mu }{a}_{\mu }$$

For the problem studied, the four boundary conditions, given by Eq. (22), are reduced to

46 $$V\left(0\right)=0$$

47 $$\frac{dV\left(0\right)}{dx}=0$$

48 $$\frac{{d}^{2}V\left(1\right)}{d{x}^{2}}=\sum_{m=0}^{n}(m+1)(m+2){a}_{m+2}=0$$

49 $$\frac{{d}^{3}V\left(1\right)}{d{x}^{3}}=\sum_{m=0}^{n}\left(m+1\right)\left(m+2\right)(m+3){a}_{m+3}=0$$

The coefficients *a*_4_-*a*_*n*_ can be simplified, with their expressions given in terms of *a*_0_ and *a*_1_

50 $${a}_{4}=\frac{({P}_{0}-2r{a}_{2}-{k}_{1}{a}_{0}-{k}_{2}{A}_{0})}{24}$$

51 $${a}_{5}=\frac{({P}_{1}-6r{a}_{3}-{k}_{1}{a}_{1}-{k}_{2}{A}_{1})}{120}$$

52 $${a}_{6}=\frac{({P}_{2}-12r{a}_{4}-{k}_{1}{a}_{2}-{k}_{2}{A}_{2})}{360}$$

53 $${a}_{7}=\frac{({P}_{3}-20r{a}_{5}-{k}_{1}{a}_{3}-{k}_{2}{A}_{3})}{840}$$

54 $${a}_{8}=\frac{({P}_{4}-30r{a}_{6}-{k}_{1}{a}_{4}-{k}_{2}{A}_{4})}{1680}$$

55 $${a}_{9}=\frac{({P}_{5}-42r{a}_{7}-{k}_{1}{a}_{5}-{k}_{2}{A}_{5})}{3024}$$

56 $${a}_{10}=\frac{({P}_{6}-56r{a}_{8}-{k}_{1}{a}_{6}-{k}_{2}{A}_{6})}{3024}$$

$$\begin{gathered} \vdots \hfill \\ a_{n} \hfill \\ \end{gathered}$$

Both coefficients *a*_0_ and *a*_1_ will be determined from Eqs. (28) and (29); finally, the approximate deflection function is obtainable via Eq. (44) as follows.

57 $$V\left(x\right)={a}_{0}+{a}_{1}x+{a}_{2}{x}^{2}+{a}_{3}{x}^{3}+{a}_{4}{x}^{4}+{a}_{5}{x}^{5}+{a}_{6}{x}^{6}+{a}_{7}{x}^{7}+{a}_{8}{x}^{8}+{a}_{9}{x}^{9}+{a}_{10}{x}^{10}+\cdots$$

## Verification and case studies

The main study focuses on Euler–Bernoulli beams with axial compression resting on linear and nonlinear elastic foundations, aiming to obtain the exact solution for the beam subjected to axial compression and transverse distributed load, which satisfies the numerical analysis system.

To validate the preceding analysis, two illustrative cases are presented in this section. In Case 1, both the applied load and the resulting deflection are intentionally prescribed as low-order polynomials, allowing the governing differential equation and boundary conditions to be satisfied exactly by a finite polynomial solution. This case is designed solely to verify the correctness of the proposed MADM formulation, including the recursive construction of Adomian polynomials, the enforcement of boundary conditions, and the associated algebraic solution procedure. The general applicability and physical relevance of MADM are subsequently evaluated in Case 2 through comparison with established results in the literature under more realistic loading conditions and nonlinear foundation behavior.

Case 1: Consider the problem with the same nondimensional governing equation and associated boundary conditions as given in Eqs. (21) to (22). The three nondimensional parameters are *k*_1_ = *k*_2_ = *r* = 1. The nondimensional applied axial compression and load are given in the following polynomial form

58 $$P\left(x\right)=\frac{8}{27}{x}^{12}-\frac{32}{9}{x}^{11}+17619{x}^{10}-\frac{1664}{27}{x}^{9}+\frac{352}{3}{x}^{8}-128{x}^{7}+64{x}^{6}+\frac{2}{3}{x}^{4}-\frac{8}{3}{x}^{3}12{x}^{2}-16x+24$$

When 17 terms are used to approximate the deflection, *N* = 17 in Eq. (44). By following the solution method as described, the 17 coefficients, *a*_0_ through *a*_16_ can satisfy the following algebraic equations:

59 $${a}_{0}=V\left(0\right)=0$$

60 $${a}_{1}={V}^{(1)}\left(0\right)=0$$

61 $$\sum_{m=0}^{N}(m+1)(m+2){a}_{m+2}={V}^{(2)}\left(1\right)=0$$

62 $$\sum_{m=0}^{N}(m+1)(m+2)(m+3){a}_{m+3}={V}^{(3)}\left(1\right)=0$$

63 $${a}_{4}=\frac{(24-2r{a}_{2}-{k}_{1}{a}_{0}-{k}_{2}{A}_{0})}{24}$$

64 $${a}_{5}=\frac{(-16-6r{a}_{3}-{k}_{1}{a}_{1}-{k}_{2}{A}_{1})}{120}$$

65 $${a}_{6}=\frac{(12-12r{a}_{4}-{k}_{1}{a}_{2}-{k}_{2}{A}_{2})}{360}$$

66 $${a}_{7}=\frac{(-\frac{8}{3}-20r{a}_{5}-{k}_{1}{a}_{3}-{k}_{2}{A}_{3})}{840}$$

67 $${a}_{8}=\frac{(\frac{2}{3}-30r{a}_{6}-{k}_{1}{a}_{4}-{k}_{2}{A}_{4})}{1680}$$

68 $${a}_{9}=\frac{(42r{a}_{7}-{k}_{1}{a}_{5}-{k}_{2}{A}_{5})}{3024}$$

69 $${a}_{10}=\frac{(64-56r{a}_{8}-{k}_{1}{a}_{6}-{k}_{2}{A}_{6})}{5040}$$

70 $${a}_{11}=\frac{(-128-72r{a}_{9}-{k}_{1}{a}_{7}-{k}_{2}{A}_{7})}{7920}$$

71 $${a}_{12}=\frac{(\frac{352}{3}-90r{a}_{10}-{k}_{1}{a}_{8}-{k}_{2}{A}_{8})}{11880}$$

72 $${a}_{13}=\frac{(-\frac{1664}{27}-110r{a}_{11}-{k}_{1}{a}_{9}-{k}_{2}{A}_{9})}{17160}$$

73 $${a}_{14}=\frac{(17619-132r{a}_{12}-{k}_{1}{a}_{10}-{k}_{2}{A}_{10})}{24024}$$

74 $${a}_{15}=\frac{(-\frac{32}{9}-156r{a}_{13}-{k}_{1}{a}_{11}-{k}_{2}{A}_{11})}{32760}$$

75 $${a}_{16}=\frac{(\frac{8}{27}-182r{a}_{14}-{k}_{1}{a}_{12}-{k}_{2}{A}_{12})}{43680}$$

As a result, these coefficients can be explicitly determined as follows:

76 $${a}_{2}=4,{a}_{3}=\frac{8}{3},{a}_{4}=\frac{2}{3}and {a}_{0}={a}_{1}={a}_{5},\cdots ={a}_{16}=0$$

Substituting these coefficients back into Eq. (21) yields the exact solution of the system, expressed as:

77 $$V(x)=4{x}^{2}-\frac{8}{3}{x}^{3}+\frac{2}{3}{x}^{4}$$

Thus, the exact solution for the deflection *V*(*x*) of an Euler–Bernoulli beam with axial compression can be obtained through this method.

As shown in Case 1, the results demonstrate that an exact solution can be derived for Euler–Bernoulli beams under axial compression when the governing equation and loading are prescribed in polynomial form. The MADM solution shows excellent agreement with the corresponding numerical results, thereby verifying the correctness of the proposed formulation. The solution procedure relies on the recursive construction of Adomian polynomials to solve the governing differential equation subject to the specified boundary and compatibility conditions. For beams resting on linear and nonlinear elastic foundations and subjected to uniform loading with axial compression, an exact polynomial solution of order $N=17$ can be obtained, yielding the displacement function $V\left(x\right)$ with coefficients ${a}_{0}$ to ${a}_{16}$. The resulting system of algebraic equations is solved using the same recursive framework inherent to the MADM formulation.

Case 2: The MADM is validated through comparison with the solutions reported by Chen et al.^15^, and this comparison is further employed to investigate the interaction between linear and nonlinear elastic foundation springs in Euler–Bernoulli beams subjected to uniformly distributed loads and axial compression. The boundary conditions considered herein are consistent with those adopted in previous Adomian-based formulations for nonlinear elastic foundation problems, which enables a direct and meaningful comparison. Nevertheless, it should be noted that further experimental investigations would be required to fully establish the physical verifiability of the model, particularly for complex foundation behaviors. For comparison purposes, the perturbation method is also employed. However, it is inherently based on the assumption of weak nonlinearity, in which the nonlinear foundation parameter $\sigma$ is treated as a small perturbation quantity. As a result, its series expansion is only valid within a restricted parameter range. When $\sigma$ exceeds this admissible range, the fundamental assumptions of the perturbation approach break down, leading to loss of convergence and the emergence of nonphysical results, such as negative deflections and excessively large errors, as observed in Table 1.

**Table 1:** Nonlinear deflection of Euler–Bernoulli beams calculated by E_*M*_ and E_*P*_ (*r* = 2.0) [E_*M*_ : MADM, E_*P*_: perturbation method].

*σ*	E_*M*_	E_*P*_	△_AVE_ (%)	E_*M*_	E_*P*_	△_AVE_ (%)	E_*M*_	E_*P*_	△_AVE_ (%)
*x*	0.1			0.5			1		
*p* = 5									
0	0.009	0.009	0.000	0.182	0.182	0.000	0.522	0.522	0.000
1	0.009	0.009	0.045	0.181	0.180	0.002	0.516	0.514	0.004
5	0.009	− 0.001	1.123	0.175	0.172	0.014	0.498	0.465	0.066
10	0.009	− 0.032	4.602	0.169	0.163	0.032	0.479	0.357	0.253
*p* = 10									
0	0.019	0.019	0.000	0.365	0.365	0.000	1.043	1.043	0.000
1	0.018	0.012	0.342	0.352	0.333	0.055	1.004	0.975	0.029
5	0.017	− 0.135	9.082	0.319	− 0.094	1.295	0.900	0.362	0.598
10	0.016	− 0.589	38.701	0.295	− 1.313	5.419	0.823	− 1.170	2.422
*p* = 20									
0	0.038	0.038	0.000	0.654	0.654	0.000	2.078	2.078	0.000
1	0.034	0.231	5.782	0.652	− 0.045	1.069	1.841	0.926	0.497
5	0.029	5.005	173.803	0.532	− 15.942	30.991	1.462	− 18.717	13.804
10	0.026	19.973	772.670	0.469	− 64.615	138.719	1.266	− 77.020	61.849

**Fig. 2:**
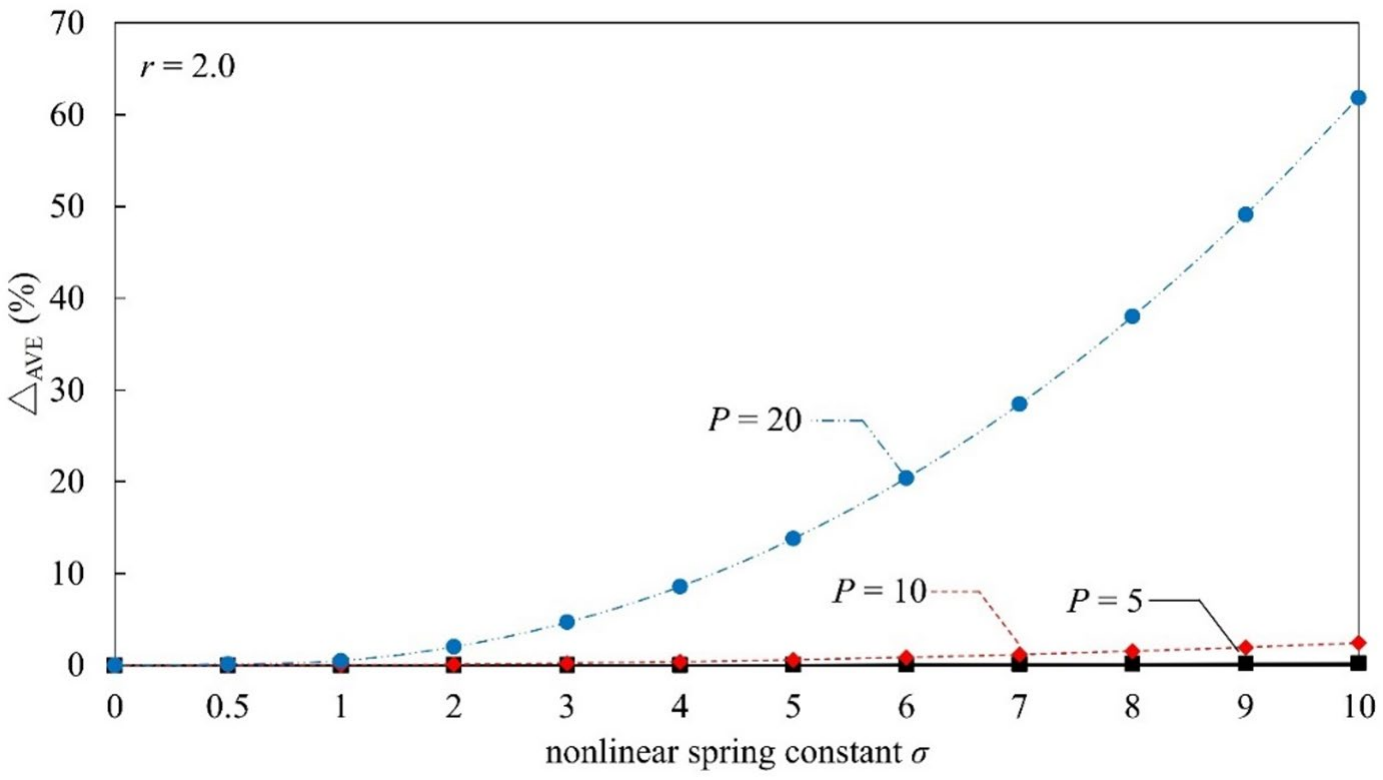
Influence of nonlinear spring constant *σ* on △AVE (%) at *r* = 2.0

Table 1 and Fig. 2 summarize the deflection results for Euler–Bernoulli beams resting on linear and nonlinear elastic foundations under axial compression and uniformly distributed loading. The average deflection at $V(1)$ is evaluated using the proposed MADM and the perturbation-based solution reported by Chen et al.^15^. To quantify the agreement between the two approaches, the relative error is defined as|△AVE(%)| =|(E_*M* _− E_*P*_)/E_*M*_ |. Here, E_*M*_ denotes the reference solution obtained by Chen et al.^15^, while E_*P*_ represents the corresponding result calculated using the perturbation method. This definition enables a clear and direct comparison between the MADM results and the perturbation-based solutions.

First, it can be inferred that when the axial compression is fixed at *r* = 2 and under a uniform load of *P* = 5, the average relative error is 0. Second, when *P* = 10, the *r* = 2, the average relative error is relatively small. Finally, when *P* = 20, *r* = 2, the average relative error increases significantly. Through the data analysis of Fig. 2 and Table 1, it can be observed that with the axial force parameter *r* fixed at 2, the larger the applied load *P* is, the more significantly the deflection of the nonlinear elastic foundation beam decreases as the nonlinear spring constant σ increases. The E_*M*_ shows that the solution converges more effectively when the nonlinear spring constant is within the range of 0–10. Conversely, the E_*P*_ indicates that the solution diverges more severely as the nonlinear spring constant deviates from this range.

These results are used to explain the influence of uniform loads and axial compression forces on nonlinear elastic foundation beams. Specifically, the beam deflection *V*(1) and the corresponding average relative error |△AVE (%)| were obtained using the E_*M*_ and the E_*P*_. A numerical method is more conducive to verifying the effectiveness of the E_*M*_ in solving linear and nonlinear elastic foundation Euler–Bernoulli beam problems. Based on the following analysis, a comparison was conducted between the E_*M*_ and the E_*P*_ regarding the nonlinear deflection *V* (σ = *k*_1_/*k*_2_ > 0). From these calculations, it can be observed that for the nonlinear elastic foundation beam (consistent with the model by Chen et al.^15^), when axial compression is applied (*r* = 2), the average deflection of both linear and nonlinear elastic foundation beams increases significantly.

## Results and discussions

For computational purposes, the MADM provides an efficient and reliable approach for solving the nonlinear governing equations of Euler–Bernoulli beams resting on elastic foundations. Through the decomposition procedure, the coefficients of the polynomial terms are determined systematically, allowing both linear and nonlinear foundation stiffness components to be treated directly without linearization. An important advantage of MADM lies in its ordered construction of polynomial terms, which promotes stable convergence as additional Adomian polynomials are included. As a result, the solution accuracy can be effectively assessed using a finite number of terms, enabling a clear and practical convergence analysis.

**Fig. 3:**
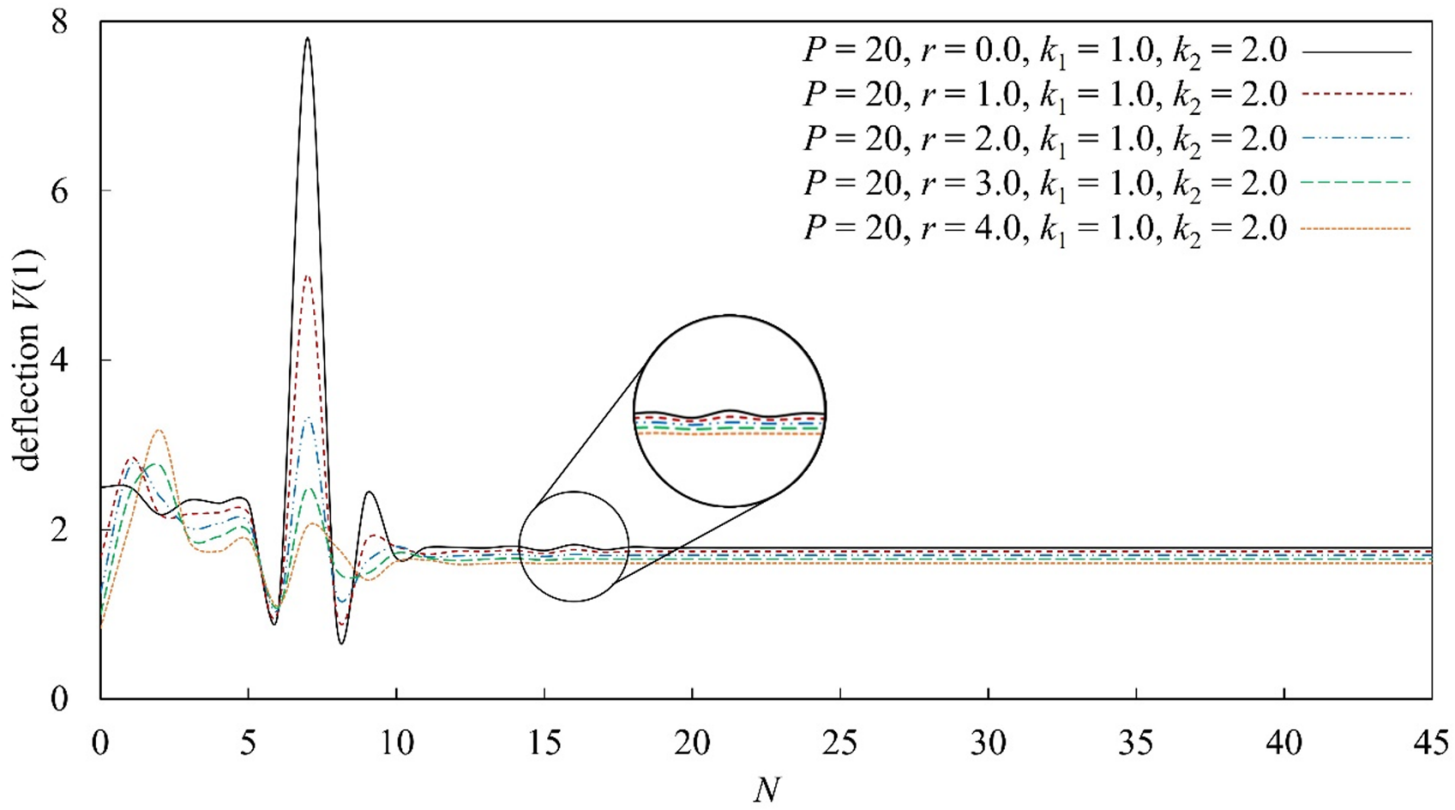
Convergence analysis of the proposed method applied to Euler–Bernoulli beams supported by linear and nonlinear elastic foundations, subjected to axial compression $r$ and a uniformly distributed load ($P=20$), with foundation stiffness parameters ${k}_{1}=1.0$ and ${k}_{2}=2.0$.

The convergence behavior of MADM for a nonlinear elastic foundation beam under axial compression is examined in Fig. 3. For the parameter set $P=20$, ${k}_{1}=1$, ${k}_{2}=2$, and $r=0$, the deflection $V\left(1\right)$ obtained with a low polynomial order ($N=7$) is relatively large, indicating that low-order truncation is insufficient to capture the contribution of the nonlinear foundation stiffness. As $N$ increases, the deflection decreases rapidly, reflecting a steep reduction in truncation error during the initial convergence stage. In the absence of axial compression, noticeable fluctuations occur for $N=15$–17, which can be attributed to the increased sensitivity of the nonlinear foundation response to higher-order Adomian polynomial terms near the convergence transition. When axial compression is introduced ($r\ne 0$), the system response becomes more regular and the convergence behavior is significantly improved. From a quantitative perspective, axial compression effectively suppresses excessive curvature, reducing the amplitude of higher-order nonlinear contributions and leading to a smoother decay of successive polynomial terms. As a result, the deflection stabilizes within the range $N=18$–45, where additional Adomian terms contribute negligibly to the solution. This convergence plateau indicates that the dominant nonlinear effects are fully captured within this range and that the truncation error falls below the prescribed tolerance. These results confirm that MADM achieves stable and reliable convergence for nonlinear beam–foundation interaction problems, particularly when axial compression is present.

**Fig. 4:**
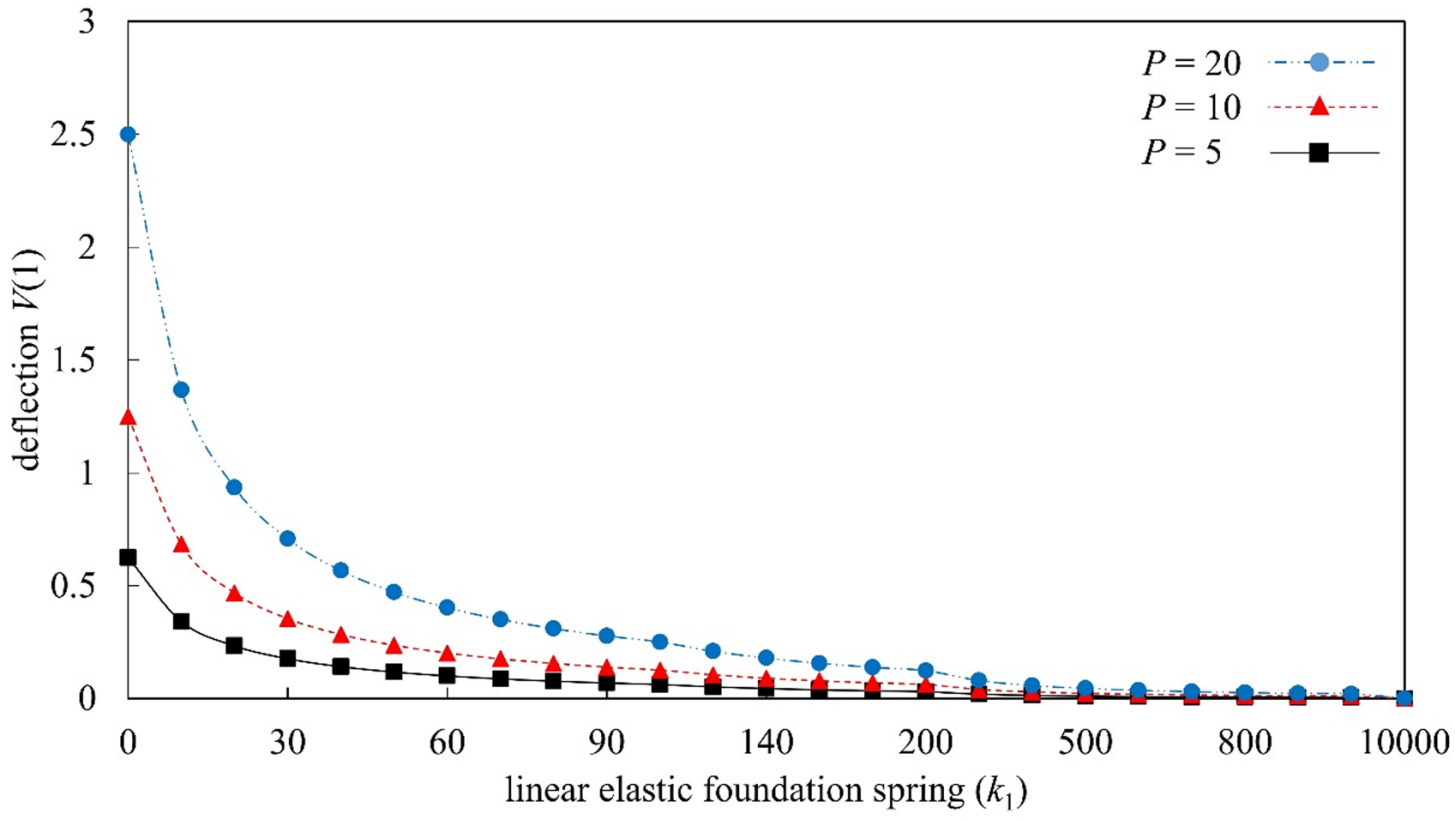
Deflection response of Euler–Bernoulli beams as a function of the dimensionless linear elastic foundation stiffness ${k}_{1}$, evaluated under the conditions $r=0.0$ and ${k}_{2}=0.0$.

**Fig. 5:**
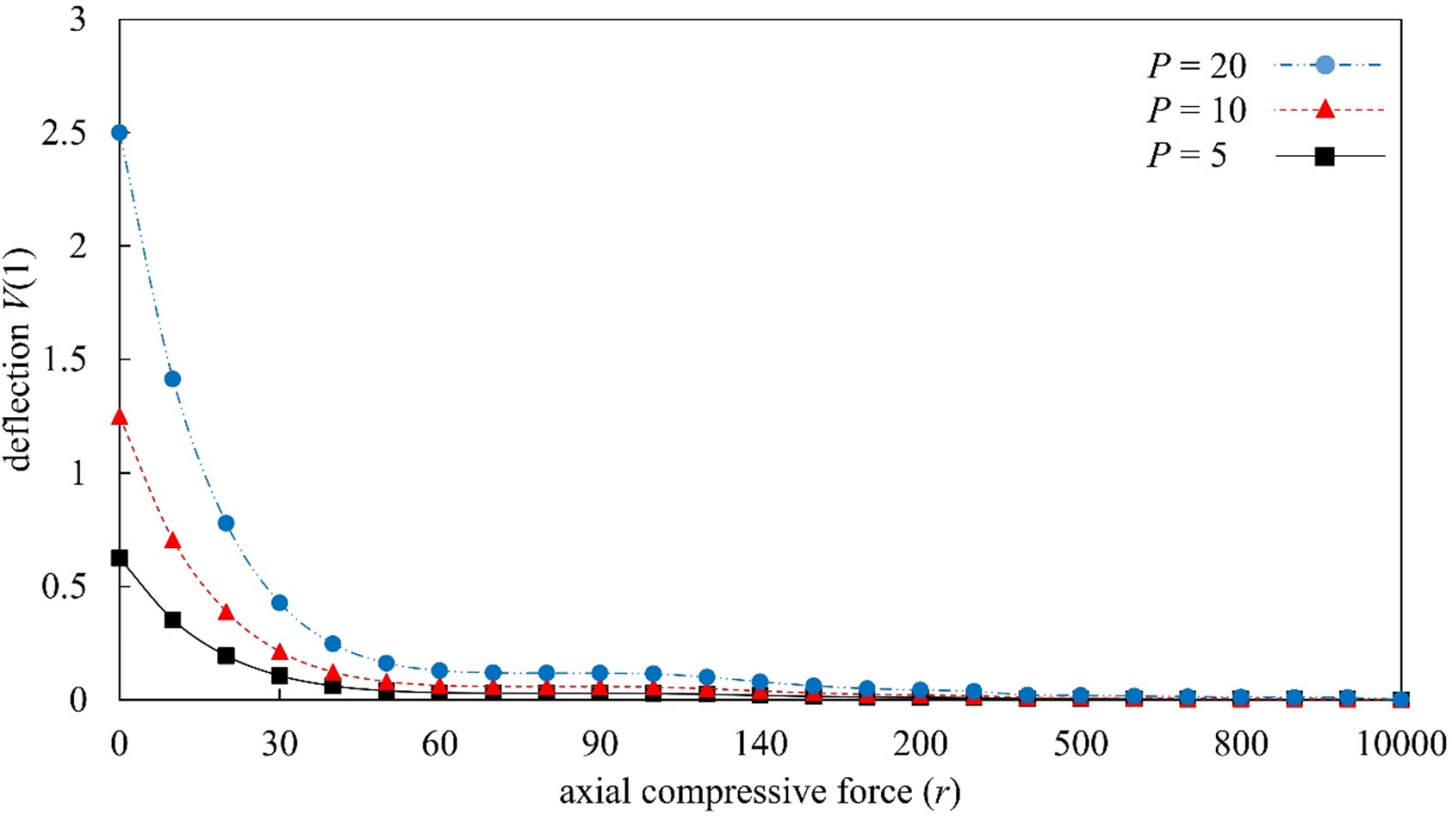
Deflection response of Euler–Bernoulli beams as a function of the dimensionless axial compression parameter $r$, evaluated under the conditions ${k}_{1}=0.0$ and ${k}_{2}=0.0$.

**Fig. 6:**
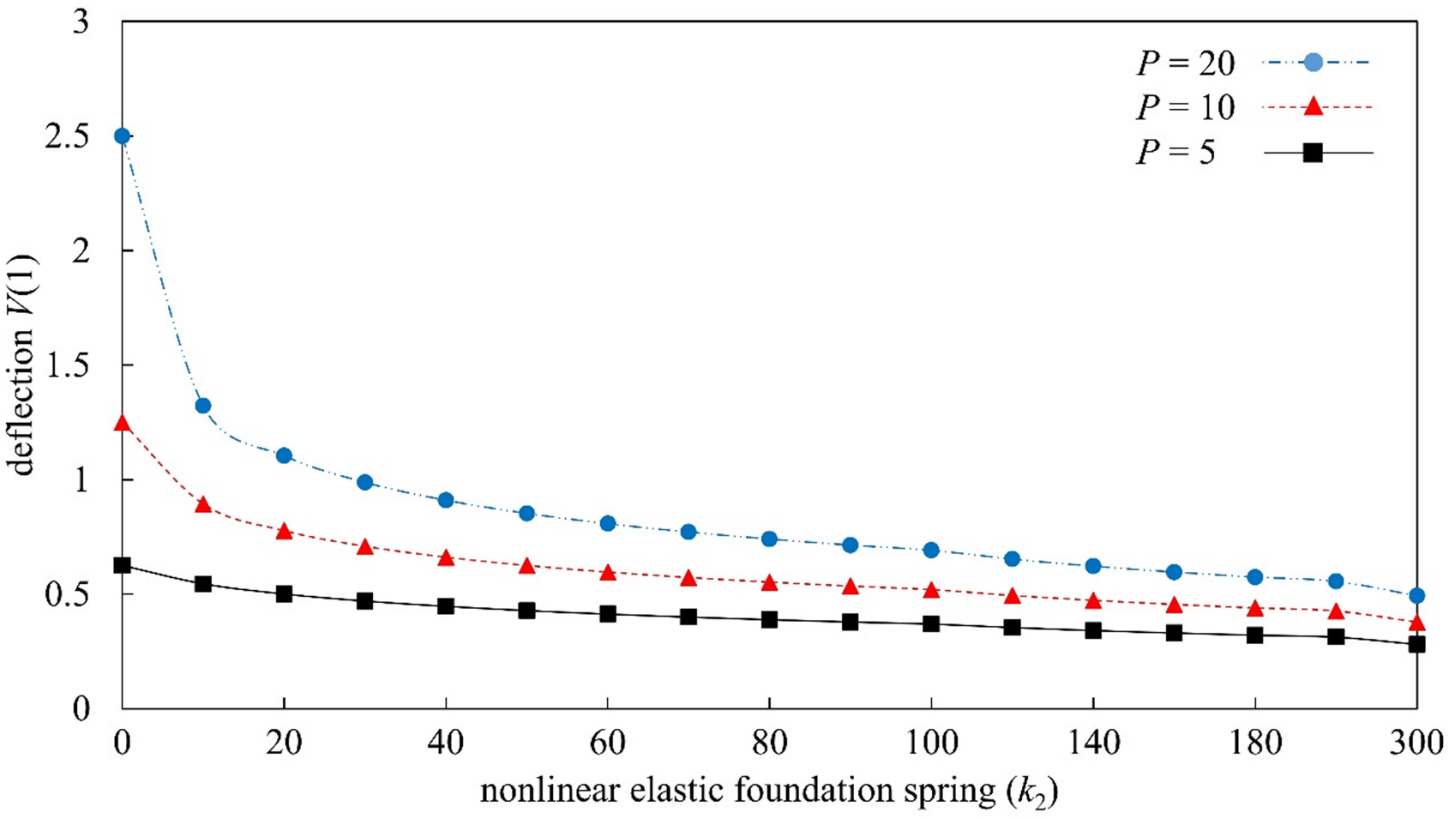
Deflection response of Euler–Bernoulli beams as a function of the dimensionless nonlinear elastic foundation stiffness ${k}_{2}$, evaluated under the conditions $r=0.0$ and ${k}_{1}=0.0$.

**Fig. 7:**
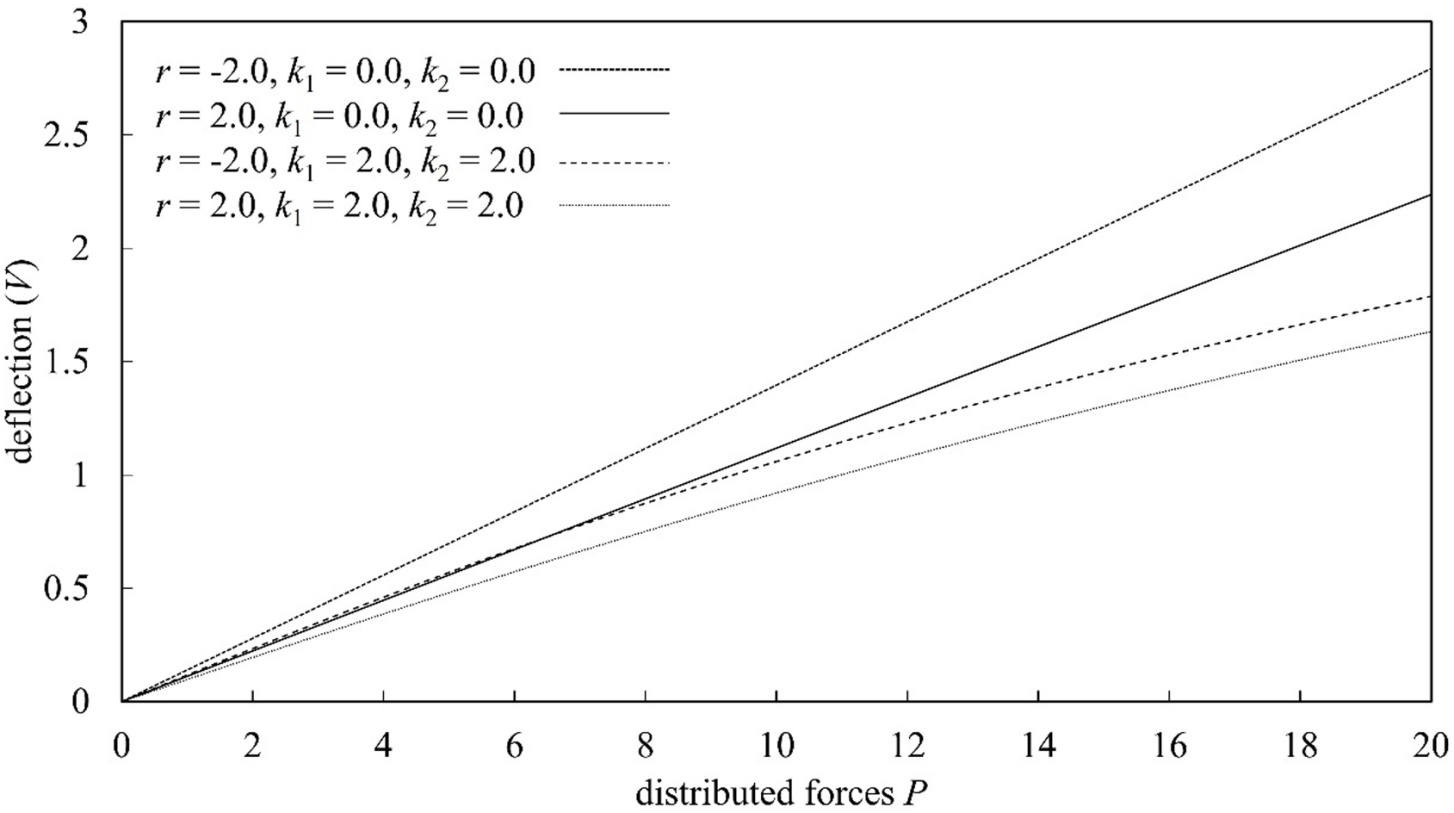
Deflection response of Euler–Bernoulli beams as a function of the dimensionless distributed load $P$, evaluated under axial compression and tension ($r=\pm 2.0$) and foundation stiffness conditions $k_{1} = 0.0,2.0$ and ${k}_{2}=0.0,2.0$.

Figures 4, 5, 6, and 7 present a parametric analysis of the deflection behavior of Euler–Bernoulli beams subjected to uniformly distributed loads, axial forces, and linear or nonlinear elastic foundations. All results are obtained using MADM, which exhibits stable convergence over the investigated parameter ranges. Figure 4 illustrates the influence of the linear elastic foundation stiffness ${k}_{1}$ in the absence of axial load ($r=0$) and nonlinear foundation effects (${k}_{2}=0$). The deflection increases monotonically with the distributed load $P$, consistent with classical bending behavior in which larger external loads induce greater bending moments. Increasing ${k}_{1}$ significantly reduces the beam deflection, as a stiffer foundation provides stronger upward resistance and suppresses bending deformation. When ${k}_{1}$ exceeds a certain level, the rate of deflection reduction becomes more gradual, indicating a diminishing marginal contribution of foundation stiffness. The influence of the axial force parameter $r$ is illustrated in Fig. 5 for beams without foundation support ($k_{1} = 0,k_{2} = 0$). The transverse deflection increases with the applied load $P$. Under axial compression ($r>0$), the deflection is reduced; however, this response should be interpreted as a pre-buckling equilibrium effect rather than a physical increase in bending rigidity. Within the framework of Euler–Bernoulli beam theory, axial compression reduces the effective bending stiffness, and the observed decrease in deflection is associated with a modified equilibrium configuration in the pre-buckling regime. A detailed investigation of behavior at or beyond the critical buckling load would require a different theoretical formulation and is beyond the scope of the present study. As $r$ increases, the rate of deflection reduction gradually diminishes, indicating that the governing equations become increasingly sensitive as the system approaches the Euler critical buckling load. All results presented here correspond to axial compression levels strictly below the critical buckling threshold, and no post-buckling behavior is considered. Conversely, axial tension ($r<0$) enhances bending deformation, resulting in larger deflections under the same applied load.

Figure 6 investigates the influence of the nonlinear foundation stiffness ${k}_{2}$ in the absence of axial loading ($r=0$) and linear foundation support (${k}_{1}=0$). The deflection increases monotonically with the distributed load $P$; however, increasing ${k}_{2}$ leads to a pronounced reduction in deflection. Quantitatively, this reduction is more significant at higher load levels, indicating the onset of nonlinear stiffening, where the restoring force increases superlinearly with displacement. As ${k}_{2}$ increases, the slope of the load–deflection response decreases progressively, reflecting enhanced resistance to bending deformation. Beyond a certain range of ${k}_{2}$, the rate of deflection reduction diminishes, suggesting a saturation threshold in the nonlinear foundation effect, where further increases in stiffness yield only marginal additional reduction in deflection. Figure 7 further analyzes the combined effects of foundation stiffness and axial force by considering $r=\pm 2.0$, ${k}_{1}=0.0$ or 2.0, and ${k}_{2}=0.0$ or 2.0. The results clearly show that when both foundation coefficients are zero (${k}_{1}={k}_{2}=0$), axial compression ($r=2$) suppresses deflection, whereas axial tension ($r=-2$) amplifies it. As the distributed load $P$ increases, the difference between the compression and tension responses becomes increasingly pronounced. When linear and nonlinear foundation stiffnesses are included (${k}_{1}={k}_{2}=2$), the combined foundation support effectively reduces deflection under axial compression. The foundation stiffness provides additional restoring force, counteracting the bending deformation amplified by compression. Under axial tension ($r=-2$), the beam remains more susceptible to deformation even when foundation support is present, and the deflection continues to grow as the load increases. The beam responses in Figs. 4, 5, 6, and 7 show consistent physical trends, the deflection increases with the load $P$, while higher foundation stiffnesses (${k}_{1}$ and ${k}_{2}$) and axial compression ($r>0$) reduce bending deformation. The nonlinear foundation is particularly effective at larger deflections due to its rapidly increasing restoring force, whereas axial tension amplifies the deflection. The results also confirm the stable and convergent performance of MADM for Euler–Bernoulli beams under axial forces and linear or nonlinear elastic foundations.

## Conclusions

This study contributes to the analysis of Euler–Bernoulli beams on nonlinear elastic foundations under axial compression by introducing an efficient solution framework based on the MADM. The proposed method directly handles nonlinearities without linearization and exhibits stable convergence as the number of Adomian polynomial terms increases, demonstrating its robustness for nonlinear beam–foundation interaction problems. The combined effects of uniformly distributed loads, axial compression, and linear and nonlinear foundation stiffness on beam deflection are systematically investigated. Comparisons with reference solutions show excellent agreement, and the MADM maintains a small and acceptable error range even under pronounced nonlinear conditions, confirming its accuracy and reliability. In addition, the proposed MADM accurately represents the nonlinear restoring behavior of the foundation springs and predicts the deflection response under various parameter combinations. Its computational efficiency and stable convergence characteristics make it suitable for nonlinear beam–foundation analyses where conventional numerical methods may exhibit limitations. Overall, the results demonstrate that MADM provides an effective and reliable analytical framework for analyzing the axial deflection of beams resting on nonlinear elastic foundations.

## Supplementary Information

Below is the link to the electronic supplementary material.

Supplementary Material 1

## Data Availability

Correspondence and requests for materials should be addressed to Ming-Xian Lin.
