# Supplementary material for: Analytic solutions for Euler–Bernoulli beams with axial compression resting on a nonlinear elastic foundation using MADM
Source: Sci Rep. 2026 Mar 3;16:13059. doi: 10.1038/s41598-026-41700-2 (PMC13099955; doi:10.1038/s41598-026-41700-2)
Supplement: Supplementary file 1 — Supplementary Material 1 [file 41598_2026_41700_MOESM1_ESM.pdf]

Analytic Solutions for Euler–Bernoulli Beams with Axial Compression Resting on a Nonlinear Elastic Foundation Using MADM

Li-Kuo Chou [1], Ming-Xian Lin [2,*]

[1]College of Optoelectronic Manufacturing, Zhejiang Industry & Trade Vocational College, 325003, Zhejiang Province, China. E-mail: love1245124512@hotmail.com
[2] Department of Mechanical and Computer-Aided Engineering, Feng Chia University, Taichung, Taiwan, R.O.C. E-mail: mxlin@o365.fcu.edu.tw
* Correspondence author

Abstract:

This article investigates the deflection of Euler-Bernoulli beams with axial compression resting on a nonlinear elastic foundation. The study evaluates the Modified Adomain Decomposition Method (MADM) for predicting beam deflection under conditions. MADM adopts an initial polynomial ansatz to optimize the nonlinear terms in the Adomian polynomials, it possesses rapid convergence and an accurate series solution. Two cases validate the method's accuracy, to verify the approach. We perform a comparative analysis based on two illustrative cases and cross-check the results with prior studies. Based on two illustrative cases, this study conducts a comparative analysis of the proposed MADM method, and cross-validation with existing research results demonstrates that its outcomes are reliable and dependable. The findings confirm that MADM provides a stable and efficient analytical framework for modeling axial compression and nonlinear foundation effects in Euler–Bernoulli beams.

Key words

Euler–Bernoulli beams, Modified Adomain Decomposition Method, nonlinear elastic foundation beam, axial compression

1. Introduction

The theory of elastic structures, particularly beams and plates resting on elastic foundations, plays a central role in modern structural mechanics due to its wide applications in helicopter blades, turbine blades, railway systems, and bridge structures. Since the early development of elastic foundation theory, extensive studies have focused on formulating governing differential equations and boundary conditions to investigate static deflection and vibration responses under various loading scenarios [1–3]. Systematic validation has been shown to improve modeling accuracy and stability [4–6]. When analyzing beams on elastic foundations subjected to uniformly distributed loads, researchers generally derive the governing differential equations along with appropriate boundary conditions to determine the beam deflection and vibration characteristics. Numerical values are then obtained through various mathematical formulations, focusing on both static and dynamic responses. Understanding the relationship between the governing variables in these equations is crucial for accurately predicting vibration frequencies and static deflections, which are

fundamental for reliable engineering applications.

Beams are often modeled with uniform geometries [7–18], and structural analysis is typically performed in a systematic and sequential manner. The Euler–Bernoulli and Timoshenko beam theories remain the classical foundations for analyzing beam behavior, and many studies have rigorously examined their accuracy and applicability [5, 9–10, 13, 15, 18, 19]. A wide variety of analytical and numerical techniques have been developed to address linear and nonlinear beam problems on elastic foundations. These include the perturbation method [4], shift function approach [8], finite element method (FEM) [19], Adomian decomposition method (ADM) [20–22], and differential transform method (DTM) [23–28]. These methods provide flexible mathematical tools for solving complex beam–foundation interactions, covering a wide range of loading, boundary, and material conditions.

In the past five years, many scholars have applied various analytical and numerical methods to investigate the behavior of beams on elastic foundations. Zhang et al. [29] compared Euler–Bernoulli and Timoshenko beam formulations to study the effects of moving loads on periodically supported beams. Their results showed that for a moving constant load expressed as an equivalent surface roughness, the influence of load speed is negligible until the sleeper passing frequency approaches the vertical resonance of the track, where the track mass begins to bounce on the support stiffness. Xu et al. [30] analyzed the transverse free vibration of an Euler–Bernoulli beam with pre-axial pressure resting on a variable Pasternak elastic foundation under arbitrary boundary conditions. They solved the governing differential equation containing nonlinear partial derivative terms of the shape function by constructing a system of stiffness equations composed of obtained matrices, instead of relying on a single equation based on classical beam theory. Doeva et al. [31] performed a static analysis of composite beams on variable stiffness elastic foundations using the Homotopy Analysis Method (HAM). Their results were compared with both published literature and the Chebyshev Collocation Method to confirm the validity and accuracy of the proposed approach. Luo et al. [32] developed an exact closed-form solution for the free vibration of Euler–Bernoulli and Timoshenko beams with intermediate elastic supports. The accuracy of their solution was verified using numerical simulations based on the Finite Element Method (FEM) and validated through comparisons with results obtained by the Transfer Matrix Method (TMM) and Green's Function Method (GFM).

Hadji et al. [33] investigated the bending and free vibration of porous functionally graded (PFG) beams resting on elastic foundations. They adopted hyperbolic shear deformation theory to formulate kinematic relations and derived the equations of motion using Hamilton's principle. Kanwal et al. [34] examined the influence of shear deformation and rotary inertia on elastically constrained beams supported by Pasternak foundations. Their findings demonstrated the precision and efficiency of the Finite Element Method through comparisons with analytical results for both general and special cases. Olotu et al. [35] performed a free vibration analysis of tapered Rayleigh beams on variable two-parameter elastic foundations and explored the effects of Pasternak foundation variation on natural frequencies for different slenderness ratios. Mellal et al. [36] analyzed the vibration and buckling behavior of porous functionally graded (FG) beams resting on variable elastic foundations using higher-order shear deformation theory. They derived the equations of motion through Hamilton's principle and obtained analytical solutions using Navier's method for simply supported FG beams. Wu et al. [37] proposed a simple and unified mesh-free approach to develop arbitrary-order Hermite shape functions for Euler–Bernoulli beam elements. This

formulation provides an explicit framework for constructing higher-order Hermite beam elements efficiently. Zemskov et al. [38] addressed the problem of unsteady vibrations in Euler–Bernoulli beams by considering relaxation effects in thermal and diffusion processes. The governing equations were derived from the general model of thermoelastic diffusion in a continuum using the variational D'Alembert principle, providing valuable insight into the coupled thermo-mechanical behavior of beam structures.

In summary, recent studies have considerably advanced the theoretical and computational understanding of beams resting on elastic foundations, yet most existing analytical and numerical approaches, including the finite element method, perturbation techniques, and transform-based solutions, still involve complicated formulations and demand significant computational effort, particularly when analyzing nonlinear foundation behavior under compressive or dynamic loads. Moreover, only limited attention has been given to the convergence stability and efficiency of analytical series solutions for nonlinear elastic foundation system. To address these limitations, this study employs the MADM to analyze the compressive deflection and convergence characteristics of beams on nonlinear elastic foundations. The proposed method establishes a simplified analytical framework that maintains high accuracy while effectively capturing nonlinear mechanical responses. Two illustrative examples are presented to validate the reliability of the approach. All symbolic derivations and numerical computations are implemented using Maple software, confirming that the Modified Adomian Decomposition Method provides accurate, stable, and computationally efficient solutions for nonlinear beam–foundation problems in engineering applications.

2. The Modified Adomian Decomposition Method

The MADM method, originally developed and proposed by Adomian [39], it is a straightforward, efficient, and reliable analytical technique specifically designed for solving nonlinear differential equations. Its main advantage lies in avoiding the complicated iterative or linearization procedures commonly encountered in traditional analytical and numerical methods. In this approach, the governing differential equation is first expressed in the following general form:

$$Fu(x) = Lu(x) + Ru(x) + Nu(x) = g(x). \tag{1}$$

In this equation, $Fu(x)$ contains both linear and nonlinear components $Lu(x) + Ru(x)$ represents the linear component of $Fu(x)$, while $Nu(x)$ is the nonlinear component. is the nonlinear ordinary differential operator, u is the physical quantity to be solved for, L is the highest-order linear differential operator, R is the remainder of the linear differential operation, and N is the nonlinear operator. The term $g(x)$ is a function.

The choice of L, the invertible linear operator, is theoretically arbitrary. However, considering the difficulty introduced in computing L^{-1} due to the presence of Green's functions, L is conventionally chosen to be the highest-order differential operator that is easily invertible.

Therefore, equation (1) can be rewritten as

$$Lu(x) = g(x) - Ru(x) - Nu(x). \tag{2}$$

Since $Lu(x)$ is an invertible operator, Eq. (2) can be expressed as

$$L^{-1}Lu(x) = L^{-1} g(x) - L^{-1}Ru(x) - L^{-1}Nu(x). \tag{3}$$

The operator Lu^{-1} is considered to be a definite integration from 0 to x. Suppose L is an nth order differential operator $L = \frac{d^n}{dx^n}$, then L^{-1} is an nth order integral operator. For boundary value problems, L^{-1} represents an indefinite integral, and the constant of integration $\Phi(x)$ is determined by

the boundary conditions. Therefore, equation (3) can be written as

$$u(x) = L^{-1} g(x) - L^{-1}Ru(x) - L^{-1}Nu(x) + \Phi(x) \quad (4)$$

In which the function $\Phi(x)$ satisfies $L\Phi(x)$. In this example, suppose L is a second-order derivative $L = \frac{d^2}{dx^2}$, then L^{-1} is a double integral operator. Thus, $\Phi(x)$ can be defined as follows

$$\Phi(x) = u(0) + x(\frac{du(x)}{dx}\Big|_{x=0}) \quad (5)$$

By substituting Eq. (5) into Eq. (4), $u(x)$ can be expressed as

$$u(x) = L^{-1}\, g(x) \; - \; L^{-1}Ru(x) \; - \; L^{-1}Nu(x) \; + \; u(0) \; + \; x(\frac{du(x)}{dx}\Big|_{x=0}) \quad (6)$$

In addition to decomposing the operator and finding their inverse operations, in the ADM, u(x) is expressed as a sum of infinitely many functions

$$u(x) = \sum_{n=0}^{\infty} u_n(x) = u_1(x) + u_2(x) + u_3(x) + u_4(x) + \cdots \quad (7)$$

Furthermore, if the nonlinear term $Nu(x)$ can be written as $f(u(x))$, a function of $u(x)$, then $Nu(x)$ can be decomposed as follows

$$Nu(x) = f(u(x)) = \sum_{n=0}^{\infty} A_n(x) = A_1(x) + A_2(x) + A_3(x) + A_4(x) + \cdots \quad (8)$$

In which A_n are the Adomain polynomials

$$A_0 = f(u_0) \quad (9)$$

$$A_1 = u_1 f^{(1)}(u_0) \quad (10)$$

$$A_2 = u_2 f^{(1)}(u_0) + \frac{1}{2!} u_1^2 f^{(2)}(u_0) \quad (11)$$

$$A_3 = u_3 f^{(1)}(u_0) + u_1 u_2 f^{(2)}(u_0) + \frac{1}{3!} u_1^3 f^{(3)}(u_0) \quad (12)$$

$$\vdots$$

$$A_n = \frac{1}{n!}\left[\frac{d^n}{dx^n} f(u_0(x))\right], n = 0,1,2,\cdots \quad (13)$$

Substituting Eq. (7) and (8) into (6), it yields

$$\sum_{n=0}^{\infty} u_n(x) = u(0) + x\left(\frac{du(x)}{dx}\Big|_{x=0}\right) + L^{-1}g(x) + L^{-1}r\sum_{n=0}^{\infty} u_n(x) - L^{-1}\sum_{n=0}^{\infty} A_n(x) \quad (14)$$

After expanding Eq. (14), the following recursive relation is obtained

$$u_0(x) = u(0) + x\left(\frac{du(x)}{dx}\Big|_{x=0}\right) + L^{-1}g(x) \quad (15)$$

$$u_n(x) = -L^{-1}R\sum_{n=0}^{\infty} u_{n-1}(x) - L^{-1}\sum_{n=0}^{\infty} A_{n-1}(x), n \geq 1 \quad (16)$$

From Eq. (16), it can be seen that each $u_n(x)$ depends only on $u_{n-1}(x)$ and its corresponding Adomian polynomials, and $u_0(x)$ is expressed solely in terms of the initial conditions. Hence, once the Adomian polynomials are determined, each $u_n(x)$ can be calculated. Finally, an infinite series solution can be obtained.

$$u_n(x) = \sum_{n=0}^{\infty} u_n(x) \quad (17)$$

In Eq. (17), as $n \to \infty$, the numerical solution becomes increasingly accurate. In practice, a partial sum of the series of polynomials is chosen to meet accuracy requirements. Numerical solutions obtained with MADM converge rapidly and achieve high accuracy.

3. Mathematical Modeling of the Euler–Bernoulli beams

Consider the deflection of Euler–Bernoulli beams on linear and nonlinear elastic foundation

subjection to axial compression and transverse distributed force shown in Fig. 1 is taken into consideration in this study

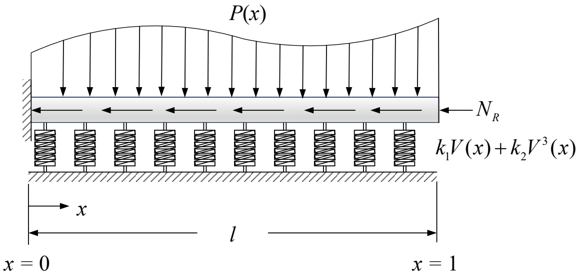

Fig. 1. A schematic diagram of an Euler–Bernoulli beam resting on a nonlinear elastic foundation subjected to axial compressive and transverse loading.

According to the formulation presented by Olotu, et. [35], the static flexural displacement $\bar{V}(X)$ of the beam is governed by the following ordinary differential equation:

$$\frac{d^2}{dX^2}\left(E(X)I(X)\frac{d^2\bar{V}(X)}{dX^2}\right) - \frac{d}{dX}\left(N_R(X)\frac{d\bar{V}(X)}{dX}\right) + K_1(X)\bar{V}(X) + K_2(X)\bar{V}^3(X) = p(X) \quad (18)$$

Here, X denotes the coordinate along the beam, and l represents the total beam length. The material and foundation properties may vary with position and are described by the Young's modulus $E(X)$, moment of inertia $I(X)$, linear foundation stiffness $K_1(X)$, and nonlinear foundation stiffness $K_2(X)$. The axial compressive force is expressed as $N_R(X)$, and the transverse load is given by $p(X)$. The boundary conditions at $X = 0$ are specified as follows:

$$EI\bar{V} = 0 \quad (19)$$

$$\frac{d}{dX}[EI\bar{V}] = 0 \quad (20)$$

at $X = 1$,

$$\frac{d}{dX}\left[EI\frac{d\bar{V}}{dX}\right] = 0 \quad (21)$$

$$\frac{d}{dX}\left[EI\frac{d^2\bar{V}}{dX^2}\right] - N_R\frac{d\bar{V}}{dX} = 0 \quad (22)$$

The nondimensional parameters for the Euler–Bernoulli beam on an elastic foundation subjected to axial compression and transverse force are defined as follows

$$x = \frac{X}{l}, V = \frac{\bar{V}(X)}{l}, P(x) = \frac{p(X)l^3}{E(X)I(X)}, k_1(x) = \frac{K_1(X)l^4}{E(X)I(X)}, k_2(x) = \frac{K_2(X)l^6}{E(X)I(X)}, r(x) = \frac{-N_R(X)l^2}{E(X)I(X)} \quad (23)$$

By substituting Eq (23) into Eqs (18) through (22), the governing differential equation can be written in the following nondimensional form:

$$\frac{d^4V}{dx^4} + r\frac{d^2V}{dx^2} + k_1V + k_2V^3 = P \quad (24)$$

and the associated boundary conditions become

at $x = 0$,

$$V(0) = 0 \quad (25)$$

$$\frac{dV(0)}{dx} = 0 \quad (26)$$

at $x = 1$,

$$\frac{d^2V(1)}{dx^2} = 0 \quad (27)$$

$$\frac{d^3V(1)}{dx^3} + r\frac{dV(1)}{dx} = 0 \quad (28)$$

Here, V denotes the transverse deflection, and x represents the spatial coordinate along the beam. The parameter k_1 is the linear elastic foundation modulus, k_2 is the nonlinear elastic foundation modulus, r refers to the axial compressive force, and Pdenotes the applied transverse load per unit length. When the applied distributed load and axial compressive force are analytic functions, the deflection $V(x)$ and the load $P(x)$can be expressed using the following Maclaurin series expansions.

$$V(x) = \sum_{m=0}^{\infty} a_m x^m \quad (29)$$

$$P(x) = \sum_{m=0}^{\infty} P_m x^m \quad (30)$$

Hence

$$\frac{dV}{dx} = \sum_{m=0}^{\infty}(m+1)a_{m+1} x^m \quad (31)$$

$$\frac{d^2V}{dx^2} = \sum_{m=0}^{\infty}(m+1)(m+2)a_{m+2} x^m \quad (32)$$

$$\frac{d^3V}{dx^3} = \sum_{m=0}^{\infty}(m+1)(m+2)(m+3)a_{m+3} x^m \quad (33)$$

$$\frac{d^4V}{dx^4} = \sum_{m=0}^{\infty}(m+1)(m+2)(m+3)(m+4)a_{m+4} x^m \quad (34)$$

The nonlinear term $V^3(x)$ can be expressed in the following form

$$V^3(x) = \{\sum_{n=0}^{\infty} a_n x^n\}\{\sum_{v=0}^{\infty} a_v x^v\}\{\sum_{\mu=0}^{\infty} a_\mu x^\mu\} = \sum_{\mu=0}^{\infty} A_m(a_0,\cdots,a_m)x^m \quad (35)$$

Where the coefficients of Adomian polynomials are

$$A_m(a_0,\cdots,a_m) = \sum_{v=0}^{m}\sum_{\mu=0}^{v} a_{m-v}a_{v-\mu} \quad (36)$$

For convenience, some coefficients of the Adomian polynomials corresponding to the nonlinear terms are listed below.

$$A_0 = a_0^3 \quad (37)$$

$$A_1 = 3a_0^2a_1 \quad (38)$$

$$A_2 = 3a_0a_1^2 + 3a_0^2a_2 \quad (39)$$

$$A_3 = 3a_0^2a_3 + a_1^3 + 6a_0a_1a_2 \quad (40)$$

$$A_4 = 3a_0^2a_4 + 3a_1^2a_2 + 6a_0a_1a_3 + 3a_0a_2^2 \quad (41)$$

$$A_5 = 3a_1^2a_3 + 3a_1a_2^2 + 3a_0^2a_5 + 6a_0a_1a_4 + 6a_0a_2a_3 \quad (42)$$

$$A_6 = 3a_0a_3^2 + 6a_0a_1a_5 + 6a_1a_2a_3 + 3a_1^2a_4 + 6a_0a_2a_4 + a_2^3 + 3a_0^2a_6 \quad (43)$$

$$A_7 = 3a_1a_3^2 + +6a_1a_2a_4 + 6a_0a_3a_4 + 3a_2^2a_3 + 3a_0^2a_7 + 6a_0a_2a_5 + 3a_1^2a_5 \quad (44)$$

$$A_8 = 6a_1a_2a_5 + 6a_0a_3a_5 + 3a_2^2a_4 + 3a_0a_4^2 + 6a_0a_2a_6 + 3a_2a_3^2 + 3a_0^2a_8 + 6a_1a_3a_4$$
$$6a_0a_1a_7 + 3a_1^2a_6 \quad (45)$$

$$A_9 = 3a_1a_4^2 + 3a_0^2a_9 + 6a_1a_2a_6 + 6a_0a_1a_8 + 6a_0a_4a_5 + 3a_2^2a_5 + a_3^3 + 6a_2a_3a_4$$
$$6a_1a_3a_5 + 6a_0a_2a_7 + 3a_2^1a_7 + 6a_0a_3a_6 \quad (46)$$

$$A_{10} = 3a_0a_5^2 + 3a_3^2a_4 + 6a_1a_2a_7 + 6a_0a_3a_7 + 6a_2a_3a_5 + 3a_0^2a_{10} + 6a_0a_4a_6$$
$$3a_2^2a_6 + 3a_1^2a_8 + 6a_0a_1a_9 + 3a_2a_4^2 + 6a_0a_2a_8 + 6a_1a_4a_5 + 6a_1a_3a_6 \quad (47)$$

$$\vdots$$

$$A_n$$

By substituting Eqs (29) through (35) back into Eq (24)

$$\sum_{m=0}^{\infty}(m+1)(m+2)(m+3)(m+4)a_{m+4}x^m + r\sum_{m=0}^{\infty}(m+1)(m+2)a_{m+2}x^m$$
$$+k_1\sum_{m=0}^{\infty} a_m x^m + k_2\sum_{m=0}^{\infty} A_m\,(a_0,\cdots,a_n)x^m = \sum_{m=0}^{\infty} P_m x^m \quad (48)$$

Once the coefficients of the power series have been collected, the following recurrence formula is obtainable.

$$a_{m+4} = \frac{P_m + R(m+1)(m+2) - k_1 a_m - k_2 A_m}{(m+1)(m+2)(m+3)(m+4)} \quad (49)$$

From the recurrence relation together with Eq. (36), it follows that all coefficients $a_m$can be expressed in terms of four unknowns, namely a_0, a_1, a_2, and a_3. These four coefficients are determined by enforcing the boundary conditions specified in Eqs. (25) - (28). Consequently, every coefficient a_m in the Maclaurin expansion of the deflection function $V(x)$ can be evaluated. In the numerical implementation, only a finite number of terms in the series is retained. When $n+1$terms are used, the approximate deflection is given by

$$V(x) = \sum_{m=0}^{n} a_m x^m \quad (50)$$

and the coefficients of Adomain polynomial are

$$A_n(a_0,\cdots,a_n) = \sum_{v=0}^{n}\sum_{\mu}^{v} a_{n=v}\, a_{v-\mu}a_{\mu} \quad (51)$$

For the problem studied, the four boundary conditions, given by Eqs (25) through (28), are reduced to

$$V(0) = 0 \quad (52)$$

$$\frac{dV(0)}{dx} = 0 \quad (53)$$

$$\frac{d^2V(1)}{dx^2} = \sum_{m=0}^{n}(m+1)(m+2)\,a_{m+2} = 0 \quad (54)$$

$$\frac{d^3V(1)}{dx^3} = \sum_{m=0}^{n}(m+1)(m+2)(m+3)\,a_{m+3} = 0 \quad (55)$$

The coefficients a_4-a_n can be simplified, with their expressions given in terms of a_0 and a_1

$$a_4 = \frac{(P_0 - 2ra_2 - k_1a_0 - k_2A_0)}{24} \quad (56)$$

$$a_5 = \frac{(P_1 - 6ra_3 - k_1a_1 - k_2A_1)}{120} \quad (57)$$

$$a_6 = \frac{(P_2 - 12ra_4 - k_1a_2 - k_2A_2)}{360} \quad (58)$$

$$a_7 = \frac{(P_3 - 20ra_5 - k_1a_3 - k_2A_3)}{840} \quad (59)$$

$$a_8 = \frac{(P_4 - 30ra_6 - k_1a_4 - k_2A_4)}{1680} \quad (60)$$

$$a_9 = \frac{(P_5 - 42ra_7 - k_1a_5 - k_2A_5)}{3024} \quad (61)$$

$$a_{10} = \frac{(P_6 - 56ra_8 - k_1a_6 - k_2A_6)}{3024} \quad (62)$$

$$\vdots$$

$$a_n$$

Both coefficients a_0 and a_1 will be determined from Eqs. (34) and (35); finally, the approximate deflection function is obtainable via Eq. (50) as follows.

$$V(x) = a_0 + a_1x + a_2x^2 + a_3x^3 + a_4x^4 + a_5x^5 + a_6x^6 + a_7x^7 + a_8x^8 + a_9x^9 + a_{10}x^{10} + \cdots \quad (63)$$

4. Verification and Case Studies

The main study focuses on Euler–Bernoulli beams with axial compression resting on linear and nonlinear elastic foundations, aiming to obtain the exact solution for the beam subjected to axial compression and transverse distributed load, which satisfies the numerical analysis system.

To validate the preceding analysis, two illustrative cases are presented in this section.

Case 1: Consider the problem with the same nondimensional governing equation and associated boundary conditions as given in Eqs (24) to (28). The three nondimensional parameters are $k_1 = k_2 = r = 1$. The nondimensional applied axial compression and load are given in the following polynomial form

$$P(x) = \frac{8}{27}x^{12} - \frac{32}{9}x^{11} + 17619x^{10} - \frac{1664}{27}x^9 + \frac{352}{3}x^8 - 128x^7 + 64x^6 + \frac{2}{3}x^4 - \frac{8}{3}x^3$$
$$12x^2 - 16x + 24 \quad (64)$$

When 17 terms are used to approximate the deflection, $N = 17$ in Eq (50). By following the solution method as described, the 17 coefficients, a_0 through a_{16} can satisfy the following algebraic equations:

$$a_0 = V(0) = 0 \quad (65)$$
$$a_1 = V^{(1)}(0) = 0 \quad (66)$$
$$\sum_{m=0}^{N}(m+1)(m+2)\, a_{m+2} = V^{(2)}(1) = 0 \quad (67)$$
$$\sum_{m=0}^{N}(m+1)(m+2)(m+3)\, a_{m+3} = V^{(3)}(1) = 0 \quad (68)$$
$$a_4 = \frac{(24 - 2ra_2 - k_1a_0 - k_2A_0)}{24} \quad (69)$$
$$a_5 = \frac{(-16 - 6ra_3 - k_1a_1 - k_2A_1)}{120} \quad (70)$$
$$a_6 = \frac{(12 - 12ra_4 - k_1a_2 - k_2A_2)}{360} \quad (71)$$
$$a_7 = \frac{(-\frac{8}{3} - 20ra_5 - k_1a_3 - k_2A_3)}{840} \quad (72)$$
$$a_8 = \frac{(\frac{2}{3} - 30ra_6 - k_1a_4 - k_2A_4)}{1680} \quad (73)$$
$$a_9 = \frac{(42ra_7 - k_1a_5 - k_2A_5)}{3024} \quad (74)$$
$$a_{10} = \frac{(64 - 56ra_8 - k_1a_6 - k_2A_6)}{5040} \quad (75)$$
$$a_{11} = \frac{(-128 - 72ra_9 - k_1a_7 - k_2A_7)}{7920} \quad (76)$$
$$a_{12} = \frac{(\frac{352}{3} - 90ra_{10} - k_1a_8 - k_2A_8)}{11880} \quad (77)$$
$$a_{13} = \frac{(-\frac{1664}{27} - 110ra_{11} - k_1a_9 - k_2A_9)}{17160} \quad (78)$$
$$a_{14} = \frac{(17619 - 132ra_{12} - k_1a_{10} - k_2A_{10})}{24024} \quad (79)$$
$$a_{15} = \frac{(-\frac{32}{9} - 156ra_{13} - k_1a_{11} - k_2A_{11})}{32760} \quad (80)$$

$$a_{16} = \frac{(\frac{8}{27} - 182 r a_{14} - k_1 a_{12} - k_2 A_{12})}{43680} \quad (81)$$

As a result, these coefficients can be explicitly determined as follows:

$$a_2 = 4,\ a_3 = \frac{8}{3},\ a_4 = \frac{2}{3} \text{ and } a_0 = a_1 = a_5, \cdots = a_{16} = 0 \quad (82)$$

Substituting these coefficients back into Eq. (24) yields the exact solution of the system, expressed as:

$$V(x) = 4x^2 - \frac{8}{3}x^3 + \frac{2}{3}x^4 \quad (83)$$

Thus, the exact solution for the deflection $V(x)$ of an Euler–Bernoulli beam with axial compression can be obtained through this method.

As shown in Case 1: the results demonstrate that we can successfully derive the exact solution of Euler-Bernoulli beams under axial compression. The MADM results show that the solution derived from this method matches the numerical solution. To obtain analytical or numerical solutions, it is essential to retrieve Adomian polynomials through recursive relationships in order to solve the governing equations. Linear and nonlinear elastic foundation beams are subjected to uniform load, and sometimes axial compression. By using the governing differential equation, boundary conditions, and boundary relational equation, the exact solution for the 17 term ($N = 17$) polynomial $V(x)$ of the beams (coefficients a_0 to a_{16}) can be obtained. Moreover, it adopts the same approach as the one used for solving the simultaneous equations in MADM.

Case 2: The MADM is validated by comparison with the solutions from Chen et al. [15], and this comparison is further used to analyze the relationship between the linear and nonlinear elastic foundation springs of Euler-Bernoulli beams under uniform distributed loads and axial compression. Additionally, this boundary condition is similar to that used in the Adomian polynomial method for deriving nonlinear elastic foundation solutions. However, its verifiability requires further validation through additional experimental studies, as illustrated below:

Table 1 and Figure 2 summarize the deflection results for beams resting on linear and nonlinear elastic foundations under axial compression and a uniformly distributed load. The average deflection at the position $V(1)$ was evaluated using two approaches: the E_M method and the formulation proposed by Chen et al. [15]. To assess the agreement between the two solutions, the relative error was calculated using $|\triangle AVE(\%)| = |(E_M - E_P)/ E_M|$.

First, it can be inferred that when the axial compression is fixed at $r = 2$ and under a uniform load of $P = 5$, the average relative error is 0. Second, when $P = 10$, the $r = 2$, the average relative error is relatively small. Finally, when $P = 20$, $r = 2$, the average relative error increases significantly. Through the data analysis of Figure 2 and Table 1, it can be observed that with the axial force parameter r fixed at 2, the larger the applied load P is, the more significantly the deflection of the nonlinear elastic foundation beam decreases as the nonlinear spring constant σ increases. The E_M shows that the solution converges more effectively when the nonlinear spring constant is within the range of 0-10. Conversely, the E_P indicates that the solution diverges more severely as the nonlinear spring constant deviates from this range.

These results are used to explain the influence of uniform loads and axial compression forces on nonlinear elastic foundation beams. Specifically, the beam deflection $V(1)$ and the corresponding average relative error $|\triangle AVE\ (\%)|$ were obtained using the E_M and the E_P. A numerical method is more conducive to verifying the effectiveness of the E_M in solving linear and nonlinear elastic

foundation Euler-Bernoulli beam problems. Based on the following analysis, a comparison was conducted between the E_M and the E_P regarding the nonlinear deflection V ($\sigma = k_1 / k_2 > 0$). From these calculations, it can be observed that for the nonlinear elastic foundation beam (consistent with the model by Chen et al. [15]), when axial compression is applied ($r = 2$), the average deflection of both linear and nonlinear elastic foundation beams increases significantly.

已註解 [明林1]: 你這段還是寫錯的阿 你是用 MADM 跟文獻 15 的微擾法結果進行比較吧? 敘述不是這個意思吧

已註解 [立C2R1]: OK

已註解 [立C3R1]:

Table 1: Nonlinear Deflection of Euler-Bernoulli Beams Calculated by E_M and E_P ($r = 2.0$) [Note: E_M : MADM, E_P : Perturbation method]

$p = 5$									
x	0.1			0.5			1		
σ	E_M	E_P	$\triangle_{AVE}$ (%)	E_M	E_P	$\triangle_{AVE}$ (%)	E_M	E_P	$\triangle_{AVE}$ (%)
0	0.009	0.009	0.000	0.182	0.182	0.000	0.522	0.522	0.000
1	0.009	0.009	0.045	0.181	0.180	0.002	0.516	0.514	0.004
5	0.009	-0.001	1.123	0.175	0.172	0.014	0.498	0.465	0.066
10	0.009	-0.032	4.602	0.169	0.163	0.032	0.479	0.357	0.253
$p = 10$									
x	0.1			0.5			1		
σ	E_M	E_P	$\triangle_{AVE}$ (%)	E_M	E_P	$\triangle_{AVE}$ (%)	E_M	E_P	$\triangle_{AVE}$ (%)
0	0.019	0.019	0.000	0.365	0.365	0.000	1.043	1.043	0.000
1	0.018	0.012	0.342	0.352	0.333	0.055	1.004	0.975	0.029
5	0.017	-0.135	9.082	0.319	-0.094	1.295	0.900	0.362	0.598
10	0.016	-0.589	38.701	0.295	-1.313	5.419	0.823	-1.170	2.422
$p = 20$									
x	0.1			0.5			1		
σ	E_M	E_P	$\triangle_{AVE}$ (%)	E_M	E_P	$\triangle_{AVE}$ (%)	E_M	E_P	$\triangle_{AVE}$ (%)
0	0.038	0.038	0.000	0.654	0.654	0.000	2.078	2.078	0.000
1	0.034	0.231	5.782	0.652	-0.045	1.069	1.841	0.926	0.497
5	0.029	5.005	173.803	0.532	-15.942	30.991	1.462	-18.717	13.804
10	0.026	19.973	772.670	0.469	-64.615	138.719	1.266	-77.020	61.849

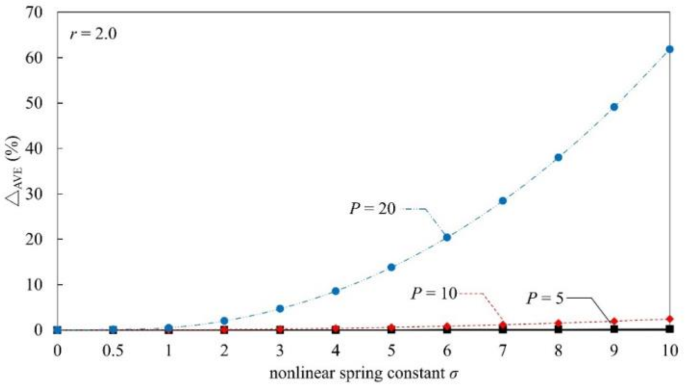

Figure 2: Influence of nonlinear spring constant σ on △AVE (%) at $r = 2.0$

5. Results and Discussions

For computational purposes, the MADM serves as an effective and powerful tool for solving the nonlinear governing equations of Euler–Bernoulli beams resting on elastic foundations. By employing the decomposition technique, the optimal convergence coefficients of the polynomial

已註解 [明林4]: 黃底幫我確認 如果確定 OK 幫我回覆確認完畢

已註解 [立C5R4]: OK

已註解 [立C6R4]:

terms can be accurately determined. This enables direct treatment of both the linear and nonlinear elastic spring components of the foundation. A key advantage of MADM is its systematic arrangement of polynomial orders, which enhances the stability of the convergence coefficients as additional Adomian polynomials are incorporated into the solution series. Consequently, the MADM framework allows the solution accuracy to be evaluated using a finite number of Adomian terms, thereby facilitating a comprehensive convergence analysis.

The convergence characteristics of MADM are further verified through the nonlinear elastic foundation beam subjected to axial compression. As illustrated in Figure 3, distinct deflection behavior is observed under various polynomial orders. Under the loading conditions of $P = 20$, $k_1 = 1$, $k_2 = 2$, and $r = 0$, the deflection $V(1)$ obtained with $N = 7$ is relatively large, primarily due to the influence of the nonlinear foundation stiffness k_2. In the absence of axial compression, the deflection exhibits noticeable fluctuations when $N$is between 15 and 17, reflecting the sensitivity of the nonlinear foundation response. However, when $r \neq 0$, the nonlinear spring stiffness $k_2$shows a clear trend toward stabilizing the system as the axial compressive force increases. This stabilizing effect enhances the effectiveness of MADM, enabling the nonlinear foundation beam response to progress toward a stable and consistent pattern. Ultimately, for N within the range of 18-45, the deflection solution demonstrates well-defined convergence. These results confirm that MADM provides reliable and logically consistent numerical performance for the nonlinear beam–foundation interaction problem under axial compression.

Figures 4 through 7 present a comprehensive analysis of the deflection behavior of Euler–Bernoulli beams subjected to uniformly distributed loads, axial forces, and linear or nonlinear elastic foundations. All results were obtained using the MADM, which consistently demonstrates stable convergence across the parameter ranges considered. Figure 4 illustrates the effect of the linear elastic foundation stiffness $k_1$under conditions without axial load ($r = 0$) and without nonlinear foundation influence ($k_2 = 0$). As expected, the deflection increases monotonically with the applied distributed load P, reflecting the classical bending response in which larger external loads induce greater internal bending moments. Increasing the linear foundation stiffness $k_1$substantially reduces the beam deflection. Physically, a larger $k_1$represents a stiffer foundation offering stronger upward resistance, effectively suppressing the bending deformation. Once k_1 exceeds a certain level, the reduction in deflection becomes more gradual, indicating the diminishing marginal effect of foundation stiffness as the system approaches its practical stiffness limit. The influence of the axial force parameter r is depicted in Figure 5 for beams without foundation support ($k_1 = 0$, $k_2 = 0$). The deflection again increases with the applied load P. However, axial compression ($r > 0$) leads to a noticeable reduction in deflection, attributed to the effective stiffening effect induced by compressive axial force. This axial stiffening reduces the curvature generated by bending moments. As r grows, the reduction in deflection gradually tapers off, illustrating the diminishing stabilizing effect of increasing compression and approaching the pre-buckling stiffness boundary predicted by Euler-Bernoulli stability theory. Conversely, axial tension ($r < 0$) increases the beam's tendency to bend and results in larger deflections.

Figure 6 examines the role of the nonlinear foundation stiffness $k_2$in the absence of axial loading ($r = 0$) and linear foundation effects ($k_1 = 0$). The results show that deflections rise with increasing load P, while increasing the nonlinear foundation parameter $k_2$significantly decreases the deflection. This behavior highlights the strengthening feature of nonlinear elastic support: the restoring force grows more rapidly with increasing deflection than in the linear case, offering

enhanced resistance under large deformation. Similar to the trends observed for k_1, the deflection reduction becomes progressively slower beyond a certain range of k_2, indicating a saturation in the foundation's nonlinear stiffening capacity. Figure 7 further analyzes the combined effects of foundation stiffness and axial force by considering $r = \pm 2.0$, $k_1 = 0.0$ or 2.0, and $k_2 = 0.0$or 2.0. The results clearly show that when both foundation coefficients are zero ($k_1 = k_2 = 0$), axial compression ($r = 2$) suppresses deflection, whereas axial tension ($r = -2$) amplifies it. As the distributed load Pincreases, the difference between the compression and tension responses becomes increasingly pronounced. When linear and nonlinear foundation stiffnesses are included ($k_1 = k_2 = 2$), the combined foundation support effectively reduces deflection under axial compression. The foundation stiffness provides additional restoring force, counteracting the bending deformation amplified by compression. Under axial tension ($r = -2$), the beam remains more susceptible to deformation even when foundation support is present, and the deflection continues to grow as the load increases. Across Figures 4–7, the beam responses follow a clear physical pattern: the deflection increases with the load P, while both foundation stiffnesses k_1 and k_2 and axial compression ($r > 0$) act to reduce the bending deformation. The nonlinear foundation is especially effective under larger deflections because its restoring force grows more rapidly with displacement. Conversely, axial tension magnifies the beam deflection and leads to a less stable response. The numerical results also show that the MADM consistently provides stable and convergent solutions, demonstrating its reliability for analyzing Euler–Bernoulli beams subject to axial forces and linear or nonlinear elastic foundations.

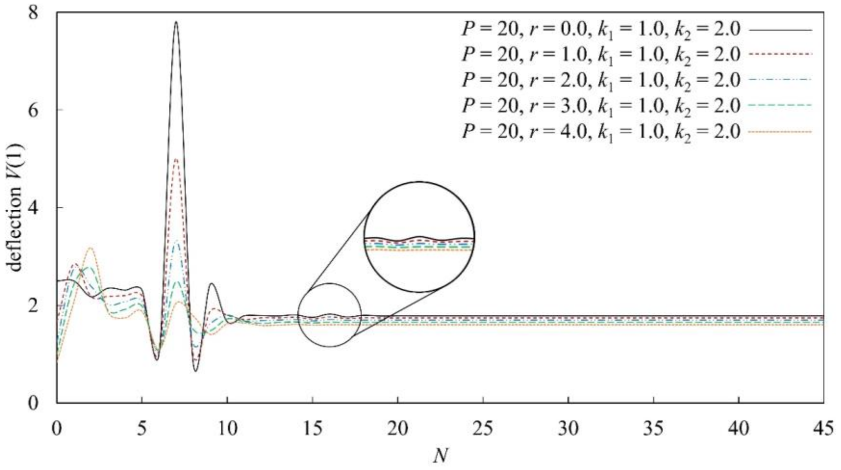

Figure 3. Convergence analysis of the proposed method applied to Euler–Bernoulli beams supported by linear and nonlinear elastic foundations, subjected to axial compression $r$and a uniformly distributed load ($P = 20$), with foundation stiffness parameters $k_1 = 1.0$ and $k_2 = 2.0$.

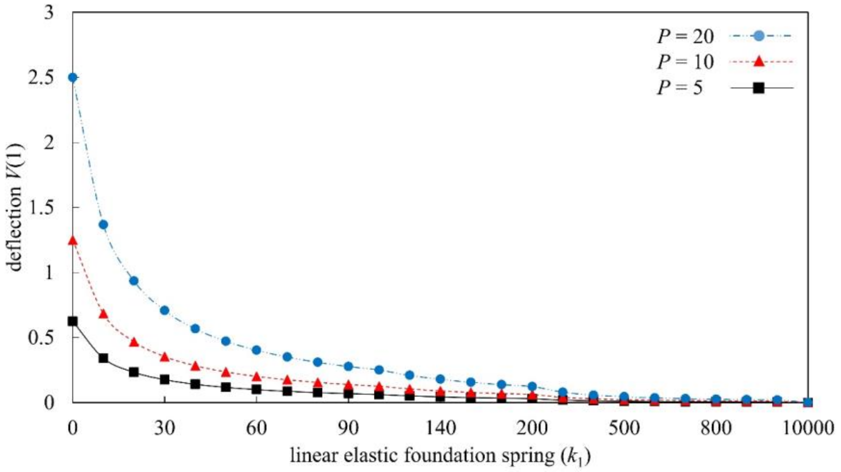

Figure 4. Deflection response of Euler–Bernoulli beams as a function of the dimensionless linear elastic foundation stiffness k_1, evaluated under the conditions $r = 0.0$ and $k_2 = 0.0$.

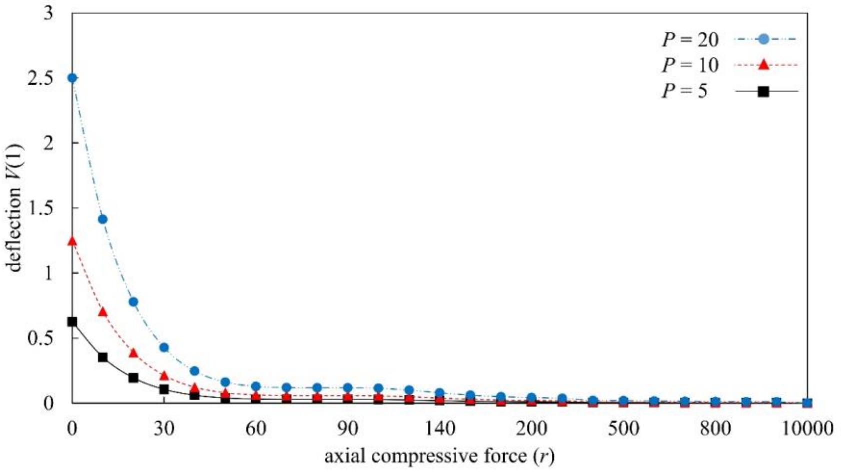

Figure 5. Deflection response of Euler–Bernoulli beams as a function of the dimensionless axial compression parameter r, evaluated under the conditions $k_1 = 0.0$ and $k_2 = 0.0$.

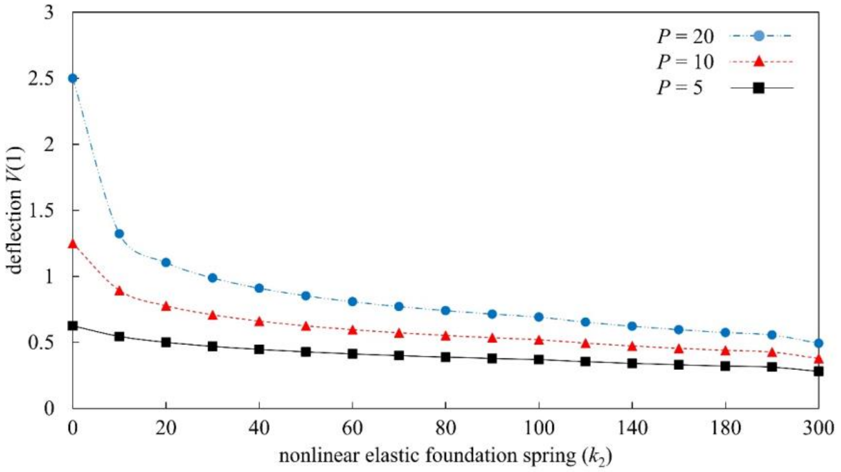

Figure 6. Deflection response of Euler–Bernoulli beams as a function of the dimensionless nonlinear elastic foundation stiffness k_2, evaluated under the conditions $r = 0.0$ and $k_1 = 0.0$.

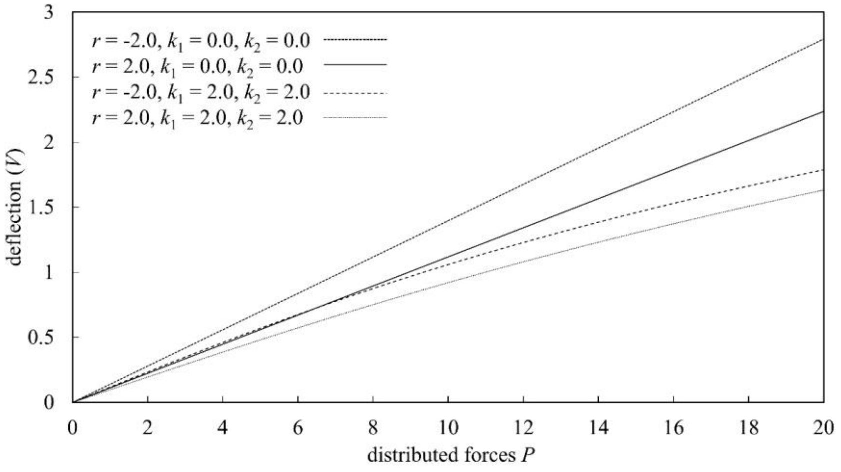

Figure 7. Deflection response of Euler–Bernoulli beams as a function of the dimensionless distributed load P, evaluated under axial compression and tension ($r = \pm 2.0$) and foundation stiffness conditions $k_1 = 0.0, 2.0$and $k_2 = 0.0, 2.0$.

6. Conclusions

In the article, this study provides a significant contribution to the analysis of Euler–Bernoulli beams resting on nonlinear elastic foundations under axial compression by introducing a reliable and efficient solution framework based on the MADM. MADM directly handles nonlinearities without linearization and demonstrates excellent numerical stability, particularly as the number of

Adomian polynomial terms Nincreases. This systematic convergence behavior establishes MADM method as a robust tool for solving nonlinear Euler-Bernoulli beam foundation interaction problems. A key contribution of this work is its comprehensive assessment of the effects of uniformly distributed loads, axial compression forces, and linear and nonlinear foundation stiffness parameters on beam deflection. The comparison between the proposed method and reference solutions shows excellent consistency. The MADM method still maintains a small and acceptable error range, confirming its accuracy in more demanding nonlinear conditions. In addition, it be successfully captured the nonlinear restoring behavior of the foundation springs and accurately predicts the deflection response under various parameter combinations. Its computational efficiency and strong convergence properties make it suitable for complex structural analyses where conventional numerical methods may exhibit limitations. Overall, the results demonstrate that MADM is a powerful and precise methodology for analyzing the axial deflection of beams on nonlinear elastic foundations, offering substantial practical value for mechanical and structural engineering applications.

Acknowledgement

This research was funded by the Ministry of Science and Technology, Republic of China, through Grant NSTC 114-2221-E-035-060, and by the major scientific research projects of Zhejiang Industry & Trade Vocational College (Grant No. yjrc202503).

Declarations

The authors declare that they have no conflict of interest.

Ethics and Consent to Participate declarations: not applicable.

Reference:

1. Lee, S. Y., & Kuo, Y. H. (1992). Exact solutions for the analysis of general elastically restrained nonuniform beams. *Journal of applied mechanics*, *59*(2S), S205-S212.
2. Kuo, Y. H., & Lee, S. Y. (1994). Deflection of nonuniform beams resting on a nonlinear elastic foundation. *Computers & structures*, *51*(5), 513-519.
3. Ho. S. H., & Chen. C. K. (1998). Analysis of general elastically end restrained nonuniform beams using differential transform, *Applied Mathematical Modelling*, 22, 219-234.
4. Huang, S. H., Lai, C. H., Lin, G. M., Yang, S. J., Cai, K. F., Lai, X. Y., & Chou, Y. L. (2025). A Data Processing Framework for Evaluating Smartphone LiDAR Accuracy and Point Cloud Correspondence. *Sensors and Materials*, *37*(11), 4919-4927.
5. Chen, J. R., Hung, S. H., & Lin, M. X. (2025). Comparison of Metrology-Grade Squareness Error Measurement Methods. *JOURNAL OF THE CHINESE SOCIETY OF MECHANICAL ENGINEERS*, *46*(1), 33-40.
6. Hsieh, T. H., Lin, M. X., & Watanabe, T. (2025). Separation of all motion errors in a rotary motor using dual rotary encoders. *International Journal of Precision Engineering and Manufacturing*, *26*(1), 117-131.
7. Girgina, Z. C., & Girgin. K. (2004). A mixed method for bending and free vibration of beams resting on a Pasternak elastic foundation, *Applied Mathematical Modelling*, 28, 877-890.
8. Lee, S. Y., Lin, S. M., Chien, S. L., Lu, S. Y., & Liu, Y. T. (2008). Exact large deflection of beams with nonlinear boundary conditions. *Computer Modeling in Engineering & Sciences*, *30*(1), 27.

9. H. A. K., Tan. T, Kaya. M. O. (2011). Free vibration analysis of beams on variable Winkler elastic foundation by using the differential transform method. *Mathematical and Computational Applications*, 16(3), 773-783.
10. Ahmed. Z., Khalid. E and Rhali. B. (2012). A homogenization procedure for nonlinear free vibration analysis of functionally graded beams resting on nonlinear elastic, *Applied Mechanic and Material*, 232, 427-431.
11. Wattansakulpong. N., & Ungbhakon. V. (2014) Linear and nonlinear vibration analysis of elastically restrained ends FGM beams with porosities. *Aerospace Science and Technology*, 32, 111-120.
12. Mirzabeigy, A., & Bakhtiari-Nejad, F. (2014). Semi-analytical approach for free vibration analysis of cracked beams resting on two-parameter elastic foundation with elastically restrained ends. *Frontiers of Mechanical Engineering*, *9*(2), 191-202.
13. Obara, P. (2014). Vibrations and stability of Bernoulli-Euler and Timoshenko beams on two-parameter elastic foundation. *Archives of Civil Engineering*, *60*(4), 421-440.
14. Rajesh, K., & Saheb, K. M. (2017). Free vibrations of uniform timoshenko beams on pasternak foundation using coupled displacement field method. *Archive of Mechanical Engineering*, *64*(3), 359-373.
15. Chen. C. K., Chou. L. K. and Lee. S. Y. (2018). Analytic static deflection solution of beams resting on strong nonlinear elastic foundations. *Journal of Chinese Society of Mechanical Engineers*, 39(1), 99-104.
16. Adair, D., Ibrayev, A., Tazabekova, A., & Kim, J. R. (2019). Free vibrations with large amplitude of axially loaded beams on an elastic foundation using the Adomian modified decomposition method. *Shock and Vibration*, *2019*(1), 3405075.
17. Kim, Y. W., & Ryu, J. Y. (2020). Vibrations of rotationally restrained Timoshenko beam at hinged supports during an earthquake. *Nuclear Engineering and Technology*, *52*(5), 1066-1078.
18. Ryu. J. Y., Kim. Y. W. (2020). Analytic responses of slender beams supported by rotationally restrained hinges during support motions. *Nuclear Engineering and Technology*, 52, 2939-2948.
19. Zhang. X., Thompson. D., Sheng. X. (2020). Differences between Euler Bernoulli and Timoshenko beam formulations for calculations for calculating the effects of moving loads on a periodically supported beam. *Journal of Sound and Vibration*. 481, 115432.
20. Chou. L. K., and Ye. Z. (2021). Analytic static deflection solutions of uniform beams resting on nonlinear elastic rotational boundary. *Journal of Mechanics Engineering and Automation*. 11, 105-113.
21. Lin, M. X., Chen, C. O. K., & Tseng, C. H. (2026). Applying Laplace Adomian Decomposition Method (LADM) for free vibration analysis of non-uniform Euler–Bernoulli beams. *Archive of Applied Mechanics*, *96*(1), 4.
22. Chou. L. K., & Lin. M. X. (2025). Analysis of Bernoulli-Euler beam on nonlinear elastic foundation using the modified Adomian decomposition method. *Engineering Computations*. DOI (10.1108/EC-06-2025-0587)
23. Lin, M. X., Tseng, C. H., & Chen, C. K. (2022). Numerical solution of large deflection beams by using the Laplace Adomian decomposition method. *Engineering Computations,* 39(3), 1118-1133.
24. Lin, M. X., Deng, C. Y., & Chen, C. K. (2022). Free vibration analysis of non-uniform Bernoulli beam by using Laplace Adomian decomposition method. *Proceedings of the Institution of Mechanical Engineers, Part C: Journal of Mechanical Engineering Science*, 236(13), 7068-7078.
25. Lin, M. X., Lee, S. Y., & Chen, C. K. (2019). Nonlocal Effect on the Pull-in Instability Analysis of

Graphene Sheet Nanobeam Actuator. *Journal of Mechanics*, 1-12.
26. Lin, M. X., Chen, C. K. (2020). Investigation of pull-in behavior of circular nanoplate actuator based on the modified couple stress theory. *Engineering computations*, 38(6), 2648-2665.
27. Lin, M. X., Lee, S. Y., & Chen, C. K. (2018). Dynamic characteristic analysis of an electrostatically-actuated circular nanoplate subject to surface effects. *Applied Mathematical Modelling*, 63, 18-31.
28. Lin, M. X., Lai, H. Y., & Chen, C. K. (2018). Analysis of nonlocal nonlinear behavior of graphene sheet circular nanoplate actuators subject to uniform hydrostatic pressure. *Microsystem Technologies*, 24(2), 919-928.
29. Zhang. X., T. David., and Sheng. X. (2020). Differential between Euler Bernoulli and Timoshenko beam formulations for calculating the effects of moving loads on a periodically supported beam. *Journal of Sound and Vibration*. 481, 115432.
30. Xu. Y., & Wang. N. (2020). Transverse free vibration of Euler Bernoulli beam with pre axial pressure resting on a variable Pasternak elastic foundation under arbitrary boundary condition. *Latin American Journal of Solids and Structures*, 17(7), e305.
31. Doeva, O., Masjedi, P. K., & Weaver, P. M. (2021). Static analysis of composite beams on variable stiffness elastic foundations by the Homotopy Analysis Method. *Acta Mechanica*, *232*(10), 4169-4188.
32. Luo. J., Zhu. S., and Zhai. W. (2022) Exact closed form solution for free vibration of Euler Bernoulli and Timoshenko beams with intermediate elastic supports. *International Journal of Mechanical Sciences*, 213, 106842.
33. Hadji, L., Bernard, F., & Zouatnia, N. (2023). Bending and free vibration analysis of porous-functionally-graded (PFG) beams resting on elastic foundations. *Fluid Dynamic and Material Process*, *19*(4), 1043-1054.
34. Kanwal, G., Nawaz, R., Ahmed, N., & Alkinidri, M. (2023). Effects of shear deformation and rotary inertia on elastically constrained beam resting on Pasternak foundation. *Physica Scripta*, *98*(6), 065017.
35. Olotu, O. T., Gbadeyan, J. A., & Agboola, O. O. (2023). Free vibration analysis of tapered Rayleigh beams resting on variable two-parameter elastic foundation. *Forces in Mechanics*, *12*, 100215.
36. Mellal, F., Bennai, R., Avcar, M., Nebab, M., & Atmane, H. A. (2023). On the vibration and buckling behaviors of porous FG beams resting on variable elastic foundation utilizing higher-order shear deformation theory. *Acta Mechanica*, *234*(9), 3955-3977.
37. Wu. Z., Wang. D., and Hou. S. (2024). A Unified Meshfree Path to Arbitrary Order Hermite Finite Elements for Euler-Bernoulli Beams. *International Journal of Structural Stability and Dynamics* 24(9), 2340029-28.
38. Zemskov. A. V., & Hao. L. V. (2025). Unsteady thermoelastic-diffusive vibrations of a Bernoulli-Euler beam on an elastic foundation. *European Journal of Mechanics / A Solids*. 113, 105707.
39. Adomian, G. (2013). *Solving frontier problems of physics: the decomposition method* (Vol. 60). Springer Science & Business Media.
